# Deficiency of a novel lncRNA-HRAT protects against myocardial ischemia reperfusion injury by targeting miR-370-3p/RNF41 pathway

**DOI:** 10.3389/fcvm.2022.951463

**Published:** 2022-09-12

**Authors:** Xinbin Zheng, Ting Zhong, Fan Yu, Jingsi Duan, Yao Tang, Yaxiu Liu, Mingrui Li, Deqiang Sun, Deling Yin

**Affiliations:** ^1^Xiangya School of Pharmaceutical Sciences, Central South University, Changsha, China; ^2^Clinical Research Center, Hainan Provincial Hospital of Traditional Chinese Medicine, Haikou, China; ^3^Department of Cardiology, The Second Affiliated Hospital, Zhejiang University School of Medicine, Hangzhou, China; ^4^Cardiovascular Key Laboratory of Zhejiang Province, Hangzhou, China

**Keywords:** lncRNA-HRAT, ischemia/reperfusion, miR-370-3p, RNF41, apoptosis, myocardial injury

## Abstract

Accumulating evidence indicates that long non-coding RNAs (lncRNAs) contribute to myocardial ischemia/reperfusion (I/R) injury. However, the underlying mechanisms by which lncRNAs modulate myocardial I/R injury have not been thoroughly examined and require further investigation. A novel lncRNA named lncRNA-hypoxia/reoxygenation (H/R)-associated transcript (lncRNA-HRAT) was identified by RNA sequencing analysis. The expression of lncRNA-HRAT exhibited a significant increase in the I/R mice hearts and cardiomyocytes treated with H/R. LncRNA-HRAT overexpression facilitates H/R-induced cardiomyocyte apoptosis. Furthermore, cardiomyocyte-specific deficiency of lncRNA-HRAT *in vivo* after I/R decreased creatine kinase (CK) release in the serum, reduced myocardial infarct area, and improved cardiac dysfunction. Molecular mechanistic investigations revealed that lncRNA-HRAT serves as a competing endogenous RNA (ceRNA) of miR-370-3p, thus upregulating the expression of ring finger protein 41 (RNF41), thereby aggravating apoptosis in cardiomyocytes induced by H/R. This study revealed that the lncRNA-HRAT/miR-370-3p/RNF41 pathway regulates cardiomyocyte apoptosis and myocardial injury. These findings suggest that targeted inhibition of lncRNA-HRAT may offer a novel therapeutic method to prevent myocardial I/R injury.

## Introduction

Ischemic heart disease is the most common type of cardiovascular disease and a primary cause of death worldwide (1, 2). Clinical treatment for myocardial infarction is effective and includes thrombolytic therapy, revascularization by percutaneous coronary intervention, or coronary artery bypass graft surgery (3). However, myocardial reperfusion induces additional death of cardiomyocytes and an increase in infarct size, a condition known as myocardial ischemia and reperfusion (I/R) injury (4–6). Over the past decades, many bioprocesses and molecular mechanisms have been shown to participate in myocardial I/R injuries, such as apoptosis, autophagy, ferroptosis, oxygen species generation, calcium overloading, and the inflammatory response (7–9). However, the mechanisms underlying myocardial I/R injury remain unclear.

As a subgroup of non-coding RNAs (ncRNAs), the general definition of long non-coding RNAs (lncRNAs) is the presence of more than 200 nt transcripts with a lack of functional coding capacity (10). Recent studies have revealed that interactions exist between lncRNAs and genetic material (DNA and RNA) as well as between their gene products (proteins), and lncRNAs regulate many genes through various mechanisms, thereby impacting diverse cellular pathways (11). LncRNAs are implicated in various pathological and physiological processes, such as cardiovascular disease and cancer (12–14). LncRNAs have been shown to regulate the initiation and progression of cardiovascular disease (15). Some lncRNAs are abnormally expressed in the heart during the early period of reperfusion after ischemia (16). Several studies have suggested that lncRNAs play a vital role in myocardial I/R injury. For example, lncRNA-H19 alleviates cardiomyocyte apoptosis and myocardial I/R injury by inhibiting the mitochondrial apoptotic pathway mediated by Bcl-2 (17). Inhibition of lncRNA-TUG1 ameliorates myocardial injury by upregulating high mobility group protein 1 (HMGB1) and Rac1 (18). In addition, upregulated lncRNA-RMRP aggravates myocardial I/R injury by downregulating miR-206 and upregulating ATG3 subsequently (19). However, the intervention by these lncRNAs only partially improved myocardial I/R injury. The existence of other lncRNAs that regulate myocardial I/R injury requires further investigation.

MicroRNAs (miRNAs) are endogenous, non-coding small RNA molecules composed of approximately 20 nucleotides. MiR-370-3p is located in the DLK1/DIO3 domain and has been identified as a cancer-related genomic region in Human chromosome 14 (20). Previous studies have shown that miR-370-3p acts as a tumor suppressor and inhibits the proliferation and invasion of various tumor cells (21–23). Recently, as direct targets of cirRNA-0010729, cirRNA-0023461, and cirRNA-TRRAP, miR-370-3p has been shown to protect cardiomyocytes from hypoxia-induced cardiomyocyte injury (24–26). However, the role of miR-370-3p in myocardial I/R injury remains unclear.

In this study, *in vitro* and *in vivo* experiments were performed to explore the role of a novel lncRNA in myocardial I/R injury and the underlying mechanism of this process. RNA sequencing analysis revealed that a novel lncRNA named lncRNA-hypoxia/reoxygenation (H/R)-associated transcript (lncRNA-HRAT) was significantly induced in H/R-cardiomyocytes and I/R-myocardium. Furthermore, we found that overexpressed lncRNA-HRAT reduced the expression of the neighboring gene adenosine A1 receptor (Adora1). In addition, lncRNA-HRAT serves as a competing endogenous RNA (ceRNA) of miR-370-3p, promoting the expression of ring finger protein 41 (RNF41), thereby aggravating apoptosis triggered by H/R in cardiomyocytes. Finally, *in vivo* studies revealed the mechanisms by which lncRNA-HRAT regulated myocardial I/R injury. In summary, these findings offer novel insights into myocardial I/R injury therapies.

## Materials and methods

### Cell culture and treatment

Cardiac muscle cell line H9c2, obtained from the Cell Bank of the Shanghai Institute of Cell Biology, Chinese Academy of Sciences, was supplemented with 100 μg/mL streptomycin, 100 U/mL penicillin G, and 10% fetal bovine serum, DMEM, and cultured at 37°C and 5% CO_2_. We built the hypoxia/reoxygenation (H/R) model as described previously (27, 28). Under an oxygen-free atmosphere (5% CO_2_, 95% N_2_, at 37°C), the cells were cultured in low glucose DMEM (1 mg/mL glucose), incubated for 8 h, and then moved into a normal culture medium (4.5 mg/mL glucose) for 48 h under a normal atmosphere (5% CO_2_ at 37°C).

LncRNA-HRAT and control lentivirus were purchased from Genechem (Shanghai, China). For lentivirus infection, after plating into 6-well plates with 30-50% confluence 24 h before use, H9c2 cells were then infected with lncRNA-HRAT lentivirus or control lentivirus at MOI = 20 for 12 h, and then placed in fresh culture medium.

### Animal

Healthy C57BL/6 male mice (weight 25-30 g, 10-12 weeks old) were purchased from the Hunan SJA Laboratory Animal Company (Changsha, China). LncRNA-HRAT floxed mice and αMyHC-Cre transgenic mice were purchased from Cyagen Biosciences (Guangzhou, China). LncRNA-HRAT floxed mice were crossed-breeding with αMyHC-Cre transgenic mice to generate cardiomyocyte-specific lncRNA-HRAT knockout (HRAT CKO) mice. Mice were kept under a 12 h light/dark cycle at 25°C, water and food were freely accessible. The Animal Research Committee of the Center of Central South University (grant 2019sydw0160) approved the animal experimental procedures, which were performed in accordance with the Guide for the Care and Use of Laboratory Animals published by the US National Institutes of Health (NIH Publication, 8th Edition, 2011).

### Myocardial ischemia/reperfusion injury model

Pentobarbital sodium (1%, 50 mg/kg intraperitoneally [i.p.]; Sigma-Aldrich, United States) was used to anesthetize age- and weight-matched male mice. Once anesthetized, the mice were intubated, and an animal ventilator (Taimeng, Chengdu, China) was used for ventilation. The left chest was opened at the fourth intercostal space, and the heart smoothly and gently “popped out” through the fourth intercostal space. 0.5 cm below the left auricle, a 6-0 silk suture was utilized to tie a slipknot around the left anterior descending coronary artery. The heart was quickly returned to the chest, the air was evacuated manually, and the skin was closed. After 45 min of ischemia, the slipknot was loosened to allow reperfusion. The sham group underwent the same procedure; however, the left coronary artery was not ligated.

### Measurement of serum creatine kinase

A creatine kinase (CK) detection kit (Nanjing Jiancheng, Jiangsu, China) was used to measure CK activity in the serum of mice, according to the manufacturer’s instructions. For each well, the absorbance was measured using a microplate reader (Tecan, Switzerland) at 660 nm.

### 2, 3, 5-triphenyl tetrazolium chloride staining

Twenty-four hours after reperfusion, the heart was resected and processed into five slices quickly. After washing with 0.9% saline, the slices were stained in the dark for 15 min with 1.0% TTC solution (Sigma-Aldrich, United States) at 37°C, and photographed. ImageJ software was used to examine the total area of the transverse section and the area of infarction, and the values obtained were averaged. The ratio of infarct size to the total area of the transverse section was defined as the infarct size (%).

### Echocardiography

Mice were anesthetized at day 7 after sham operation or I/R by isoflurane and echocardiography was performed utilizing a Vevo2100 high-resolution imaging system accompanied by a 30-MHz high-frequency transducer (FUJIFILM Visual Sonics, Canada) as described previously (29). The left ventricular (LV) internal diameter was measured for at least three beats in two-dimensional M-mode and then averaged. Left ventricular end-diastolic diameter (LVEDD) and left ventricular end-systolic diameter (LVEDS) were examined, and matched software was utilized to compute the left ventricular fractional shortening (LVFS) and left ventricular ejection fraction (LVEF).

### RNA sequencing analysis

RNA sequencing experiments were performed by RiboBio Company (Guangzhou, China). In brief, samples (two H/R treatment and two normal oxygen groups) were purified and subjected to cDNA synthesis, and subsequently underwent adaptor ligation and amplification using NEBNext^®^ Ultra™ RNA Library Prep Kit for Illumina (NEB, United States), as per manufacturer’s instructions. After assessment with the Agilent 2200 TapeStation and Qubit^®^2.0 (Life Technologies, United States), the purified library products were diluted to 10 pM to generate clusters on the pair-end flow cell *in situ*, and then sequenced (2 × 150 bp) using Illumina HiSeq3000.

After removing the low-quality reads with adapters and ploy-N from the raw data, HISAT2 was used to map the clean reads to the reference genome of Rat (rn6). Subsequently, the mapped short reads were converted to read counts via HTSeq. Using the read counts as input, DEseq was applied to evaluate differential expression. Multiple testing correction with the Benjamini-Hochberg approach was adopted. For differentially expressed genes, the thresholds for identification were adjusted to *p*-value less than 0.05 and fold change more than 2.

### MiRNA reagents and their application

The miRNA mimics were purchased from GenePharma (Shanghai, China). RiboFect™ CP Reagent (RiboBio, Guangzhou, China) was used for *in vitro* transfection of miRNA mimics. H9c2 cells were developed in 6-well plates with 30-50% confluence 24 h before use and then transfected with miRNA mimic, as per manufacturer’s instructions. After transfection for 48 h, further assays were performed.

### Luciferase reporter assay

The mutant and wild-type (WT) regions of lncRNA-HRAT and RNF41, accompanied by assumed sites binding with miR-370-3p, were synthesized, followed by construction into the GP-miRGLO-control reporter vector (Genepharma, Shanghai, China). HEK-293T cells were plated into 12-well plates 24 h before transfection, and the corresponding plasmids, miR-370-3p or NC mimics, were used for co-transfection. After 48 h, the dual luciferase reporter assay kit (Promega, Madison, United States) was used, as per manufacturer’s instructions, to test the activities of firefly and Renilla luciferase. The activity of firefly luciferase was standardized to that of Renilla luciferase, as described previously (30).

### RNA immunoprecipitation assay

RIP assay was performed using the Magna RNA-Binding Protein immunoprecipitation kit (Millipore, MA, United States), as per manufacturer’s instructions. Briefly, after transfection for 48 h with miR-370-3p or NC mimic, H9c2 cells were harvested, resuspended in RIP lysis buffer, and incubated for 5 min on ice. The cell extract and magnetic beads conjugated with 5 μg of Ago2 antibody (Cell Signaling Technology, 2897S) or IgG antibody (Millipore, PP64B) were incubated together. Rabbit IgG was used as the negative control. Magnetic beads were resuspended in the washing buffer. To separate the RNA-protein complexes from the beads, proteinase K was incubated with magnetic beads for 30 min at 55°C. After RNA extraction, lncRNA-HRAT expression in immunoprecipitated RNA was determined using qRT-PCR.

### RNA pulldown assay

The biotinylated HRAT probe was synthesized by the RiboBio Company (Guangzhou, China). After washing with cold PBS, approximately 3 × 10^7^ H9c2 cells were lysed using RIP lysis buffer. Subsequently, 1 μg of probe was added to 50 μL prewashed streptavidin beads, blended lightly, and incubated for 2 h at 4°C. The lysates were incubated overnight with probe-coated beads at 4°C. After binding to the beads, the RNA complexes were eluted and subjected to qRT-PCR to measure the miR-370-3p expression.

### Assessment the protein-coding potential of lncRNA-HRAT

To analyze the possibility of lncRNA-HRAT coding, we employed the Coding Potential Calculator Online software as described previously (11, 31). The lncRNA-HRAT sequence was used to automatically calculate its coding potential capacity, and the mRNA of TP53 and β-actin was regarded as the positive control of the coding gene (data not shown).

### Nuclear and cytoplasmic RNA isolation

To explore the cell location of lncRNA-HRAT, cytoplasmic and nuclear fractions were isolated and collected using the PARIS Kit (Life Technologies, CA, United States), according to the manufacturer’s instructions. The extracted RNA was analyzed by qRT-PCR to detect the expression of the cytoplasmic control transcript GAPDH and β-actin, nuclear control transcript U6, and lncRNA-HRAT.

### RNA fluorescent *in situ* hybridization analysis

RNA FISH was performed using a FISH Kit (RiboBio, Guangzhou, China) with probes targeting lncRNA-HRAT and probes targeting r-18S, following the manufacturer’s protocol.

### Flow cytometry analysis

The Annexin V-FITC Apoptosis Detection Kit (BD Biosciences, CA, United States) was used to perform flow cytometry to measure the apoptotic rate. Briefly, after harvest, the floating and attached cells were washed with cold PBS, resuspended in Annexin V binding buffer, stained with Annexin V-FITC and PI, and then analyzed by flow cytometry. The flow cytometer (BD Biosciences, CA, United States) was used to measure 1 × 10^6^ cells for each experiment, and Flow Jo v10.2 (BD Biosciences, CA, United States) was used for analysis. The impact of lncRNA-HRAT on apoptosis triggered by H/R was tested using Annexin V-APC and 7-AAD (BD Biosciences, CA, United States). The methods used were similar to those used for the Annexin V-FITC Apoptosis Detection Kit.

### RNA extraction and qRT-PCR analysis

Total RNA was extracted from the cultured cells and mouse heart tissues using TRIzol reagent (Invitrogen, Carlsbad, CA, United States), as per manufacturer’s instructions. Briefly, a Reverse Transcription Kit (Takara, Dalian, China) was used to reverse transcription of 1,000 ng total RNA to cDNA. For miRNA, based on the manufacturer’s instructions and our published papers, a miRNA cDNA Synthesis Kit (Cwbio, Beijing, China) was used for reverse transcription of 2,000 ng of total RNA to cDNA (28, 32). Following the manufacturer’s instructions, the cDNA obtained was subjected to real-time PCR using SYBR Green (Bio-Rad, Hercules, California, United States). For miRNA, following the instructions of the manufacturer and our previous studies, the resulting cDNA was subjected to real-time PCR using the miRNA qPCR Assay Kit (Cwbio, Beijing, China) (28, 32). GAPDH and U6 were used as normalized controls. The fold-change was computed via the 2^–ΔΔCt^ analytic approach. The remaining primers used are listed in Supplementary Table 1.

### Western blot assay and antibodies

RIPA lysis buffer (Beyotime, Shanghai, China) was used to obtain total proteins. Loaded on a 12% SDS-PAGE gel, a 30 μg protein sample was then transferred onto 0.22 μm polyvinylidene fluoride membrane (Millipore, MA, United States). After blocking with 5% milk at RT, the membrane was then incubated with antibodies against β-actin (1:5000, Proteintech, 20536-1-AP), GAPDH (1:1000; Proteintech, 10494-1-AP), Caspase-3 (1:1000, CST, 9662S), cleaved Caspase-3 (1:1000, CST, 9664S), RNF41 (1:1000, Proteintech, 17233-1-AP) overnight at 4°C. β-actin and GAPDH antibody was served as control. An enhanced chemiluminescent detection kit (NCM Biotech, Suzhou, China) was used to visualize the membranes, and image software (Version 5.1, Bio-Rad, CA, United States) was used to perform the densitometric analysis.

### Statistical analysis

The data are presented as the mean ± SD. Student’s t-test (two-tailed) was applied to analyze the data between groups, and one-way analysis of variance (ANOVA) followed by Tukey’s *post hoc* test was adopted for analysis among multiple groups. *P* value < 0.05 was significant statistically. SPSS analyses were performed using analysis 23.0.

## Results

### H/R induces lncRNA-HRAT expression

**FIGURE 1:**
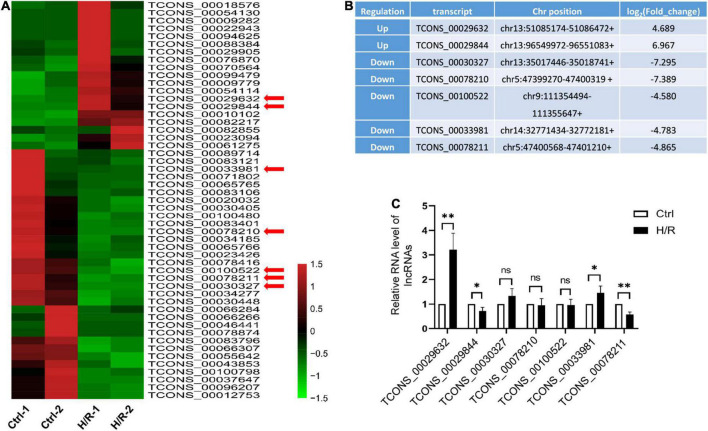
H/R alters the expression of lncRNAs. **(A)** Heat map of RNA sequencing results of dysregulated lncRNAs (fold change > 3) in Ctrl and H/R cells. **(B)** Seven novel lncRNAs with the most obvious alterations in H/R-treated H9c2 cells. **(C)** The expression of seven selected lncRNAs in Ctrl and H/R cells were analyzed by qRT-PCR (*n* = 5). The Data are presented as means ± SD. *P < 0.05, **P < 0.01, ns = not significant.

To determine which lncRNAs may contribute to cardiomyocyte injury caused by H/R, H9c2 cardiomyocytes were treated with or without H/R, and RNA sequencing analysis was performed. 51 differentially expressed lncRNAs were detected at a threshold of fold change > 3 (Figure 1A). Among these lncRNAs, 19 were upregulated and 32 were downregulated. We selected 7 lncRNAs (Figure 1B) accompanied by the following properties: (a) expression fold change > 4.5 after H/R treatment. (b) No overlap or splicing with any coding gene existing in the Ensembl genome browser annotations, University of California Santa Cruz Genome Browse, and the National Center for Biotechnology Information’s RefSeq database. The expression of the selected lncRNAs was verified using qRT-PCR (Figure 1C). Similar results for the two lncRNAs were obtained from the RNA sequencing analysis. Among the two most differentially expressed lncRNAs, a novel lncRNA TCONS_00029632 was significantly upregulated during H/R. We named this lncRNA-Hypoxia/reoxygenation-associated transcript (lncRNA-HRAT). Therefore, lncRNA-HRAT was selected for further study.

### Overexpression of lncRNA-HRAT promotes H/R-induced apoptosis in cardiomyocyte

**FIGURE 2:**
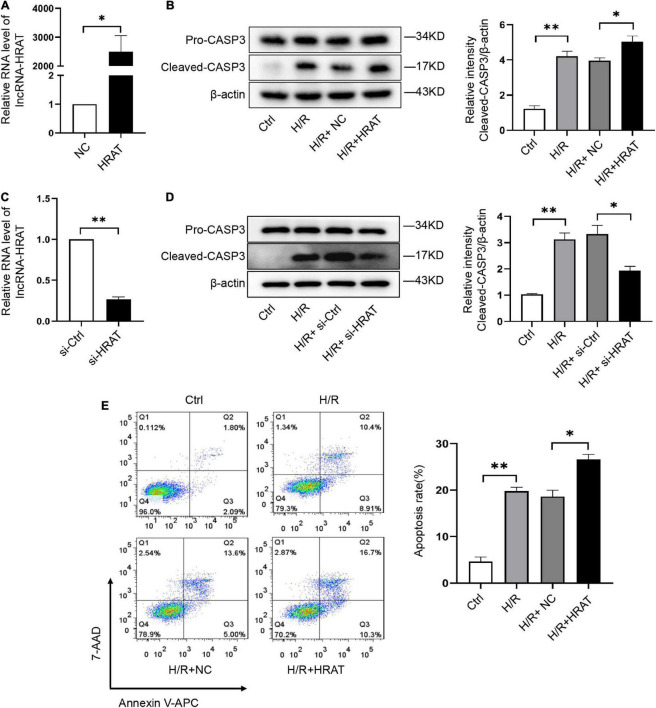
LncRNA-HRAT overexpression exacerbates the apoptosis induced by H/R in cardiomyocytes. **(A)** The analysis of lncRNA-HRAT expression in H9c2 cells infected with lncRNA-HRAT or control lentivirus was conducted through qRT-PCR (*n* = 3). **(C)** The analysis of lncRNA-HRAT expression in H9c2 cells transfected with lncRNA-HRAT or control siRNA was conducted through qRT-PCR (*n* = 3). **(B,D)** Protein levels of caspase-3 and cleaved-caspase-3 in different groups of cells were tested via Western blot (*n* = 3). **(E)** The apoptotic cell rate in H9c2 cells was measured through flow cytometry (*n* = 3). The data are described as mean ± SD. *P < 0.05, **P < 0.01.

To study the role of lncRNA-HRAT in H/R-induced cardiomyocyte injury, we used lncRNA-HRAT-overexpressing lentivirus to increase the expression of lncRNA-HRAT. We found that when infected with lncRNA-HRAT-overexpressing lentivirus, the expression of lncRNA-HRAT was significantly raised (Figure 2A). Furthermore, in comparison to the control H/R group, overexpression of lncRNA-HRAT dramatically enhanced cleaved-caspase-3 levels (Figure 2B). In contrast, knockdown of the lncRNA-HRAT significantly inhibited the expression of cleaved-caspase-3 (Figures 2C,D). We then performed flow cytometric analysis to detect apoptosis. Compared to the control H/R group, the percentage of apoptotic cells was significantly increased in the lncRNA-HRAT H/R group (Figure 2E). In summary, these data suggest that lncRNA-HRAT promotes H/R-induced apoptosis of cardiomyocytes.

### Characterization of lncRNA-HRAT

**FIGURE 3:**
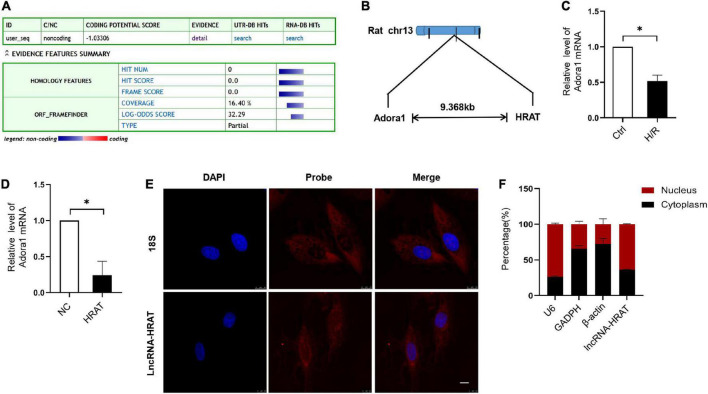
Characterization of lncRNA-HRAT. **(A)** Assessment the protein-coding potential of lncRNA-HRAT by Coding Potential Calculator Online. **(B)** Schematic annotation of the lncRNA-HRAT genomic locus. **(C)** The analysis of Adora1 expression in Ctrl and H/R cells was conducted through qRT-PCR (*n* = 3). **(D)** The analysis of Adora1 expression in H9c2 cells infected with lncRNA-HRAT or control lentivirus was conducted through qRT-PCR (*n* = 3). **(E)** The localization of lncRNA-HRAT in H9c2 cells was tested via RNA FISH (*n* = 3). Nuclei was stained with 40,6-diamidino-2-phenylindole (DAPI; blue), 18S and lncRNA-HRAT appeared red. Scale bar, 10μm. **(F)** The analysis of lncRNA-HRAT expression in the nucleus and cytoplasm of H9c2 cells was employed through qRT-PCR (*n* = 3). The cytoplasmic control was GAPDH and β-actin, while the nuclear control was U6. All the data are described as the mean ± SD. *P < 0.05.

Our coding potential calculator online analysis revealed that lncRNA-HRAT had no coding potential (Figure 3A). LncRNA-HRAT resides on chromosome 13q13 and is localized near the coding gene adenosine A1 receptor (Adora1) (Figure 3B). It has been reported that lncRNAs have cis-regulatory effects on neighboring genes (33). Thereafter, we evaluated whether lncRNA-HRAT would have an impact on the expression of nearby genes in cis. Notably, Adora1 expression was significantly downregulated during H/R (Figure 3C). Overexpression of lncRNA-HRAT dramatically decreased Adora1 expression (Figure 3D), revealing that lncRNA-HRAT affected the expression of nearby genes in cis. Subsequently, we determined the location of the lncRNA-HRAT in H9c2 cardiomyocytes. FISH and qRT-PCR analyses indicated that lncRNA-HRAT was present in both the nucleus and the cytoplasm (Figures 3E,F). Therefore, lncRNA-HRAT interacts with cytoplasmic molecules or proteins.

### Cardiomyocyte-specific deficiency of lncRNA-HRAT alleviates myocardial I/R injury in mice

**FIGURE 4:**
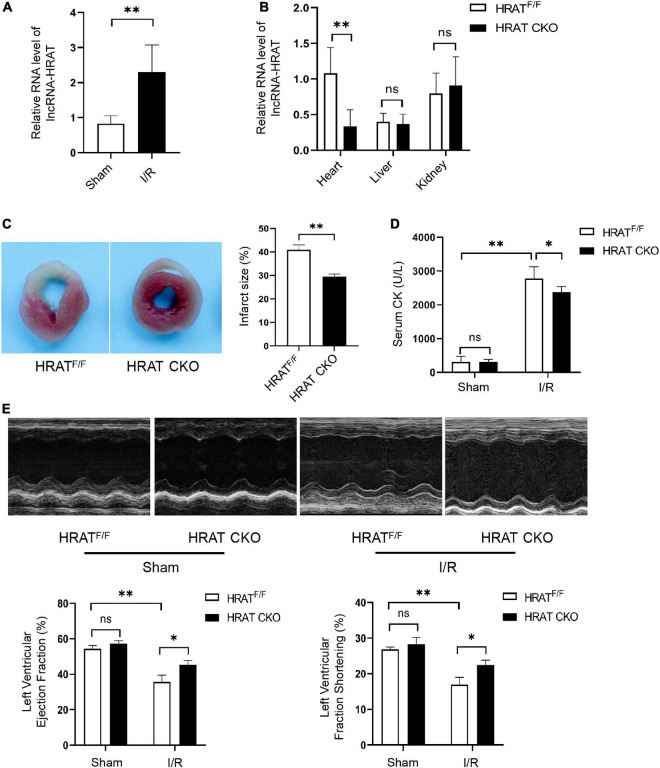
Cardiomyocyte-specific deficiency of lncRNA-HRAT protects mice hearts against I/R injury. **(A)** LncRNA-HRAT expression in I/R (45’/3 hr) and sham mice hearts was analyzed through qRT-PCR (*n* = 5). **(B)** Heart, liver, kindey were prepared from control HRAT floxed (HRAT^F/F^) and HRAT CKO mice and subjected to qRT-PCR (*n* = 5). **(C)** The infarct size of the hearts in HRAT^F/F^ and HRAT CKO mice subjected to I/R (45’/24 hr) was measured by TTC staining (*n* = 5). **(D)** Circulating levels of creatine kinase (CK) in HRAT^F/F^ and HRAT CKO mice after I/R (45’/3 hr) (*n* = 5). **(E)** Representative echocardiograms (upper) and quantitative analysis of LVEF (%) (lower left) and LVFS (%) (lower right) in diverse groups of mice at day 7 (*n* = 5). All the data are described as the mean ± SD. *P < 0.05, **P < 0.01, ns = not significant.

To detect the possible participation of lncRNA-HRAT in myocardial I/R injury, a myocardial I/R model in mice was built, and qRT-PCR was applied to test the expression of lncRNA-HRAT. The results revealed that lncRNA-HRAT was markedly increased in mouse hearts in the model (Figure 4A), indicating that lncRNA-HRAT may contribute to myocardial I/R injury. To investigate the role of lncRNA-HRAT in myocardial I/R injury, we crossed breeding lncRNA-HRAT floxed mice with α-MyHC-Cre transgenic mice to obtain cardiomyocyte-specific lncRNA-HRAT knockout (HRAT^flox/flox^; Cre^α^ ^MyHC^ (HRAT CKO)) mice. The strategy for generating HRAT CKO mice is shown in Supplementary Figure 3. The qRT-PCR results confirmed that lncRNA-HRAT was significantly reduced in the hearts of HRAT CKO mice but not in other tissues tested (Figure 4B).

To assess the role of lncRNA-HRAT in myocardial I/R injury, HRAT CKO and HRAT^flox/flox^ mice underwent ischemia for 45 min and reperfusion for 24 h. In addition, the myocardial infarct area was evidently decreased in HRAT CKO mice compared to HRAT^flox/flox^ mice (Figure 4C). The release of creatine kinase (CK) in the serum, an index of myocardial injury, was also significantly decreased in HRAT CKO mice (Figure 4D). To evaluate whether lncRNA-HRAT altered cardiac function following ischemia/reperfusion, we subjected mice to I/R and assessed cardiac function using echocardiography. Our results showed that lncRNA-HRAT knockout significantly improved LVEF (%) and LVFS (%) in I/R injured mice (Figure 4E). These results revealed that lncRNA-HRAT deficiency protects against myocardial I/R injury.

### LncRNA-HRAT directly regulates miR-370-3p

New evidence suggests that cytoplasmic lncRNAs modulate the function of miRNAs by serving as ceRNAs. Thereafter, we analyzed the potential interacting miRNAs of lncRNA-HRAT using RNAhybrid, Miranda, and PITA. The results showed that 16 miRNAs formed complementary base pairs with lncRNA-HRAT (Supplementary Table2). The qRT-PCR data implied that miR-3473 and miR-370-3p were markedly decreased in cardiomyocytes after H/R (Supplementary Figure 4A). Moreover, overexpression of lncRNA-HRAT markedly decreased miR-370-3p expression but not miR-3473 (Supplementary Figure 4B). Therefore, miR-370-3p was selected for subsequent experiments.

**FIGURE 5:**
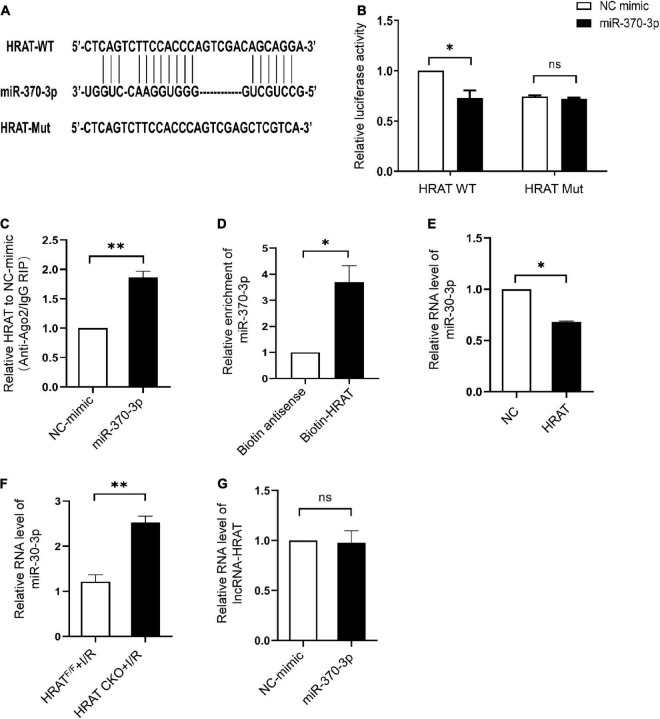
LncRNA-HRAT targets on miR-370-3p directly. **(A)** Predicted binding site between lncRNA-HRAT and miR-370-3p. **(B)** The relative activity of luciferase in the lncRNA-HRAT wild-type/mutant reporter in HEK-293T cells, with or without miR-370-3p mimics was analyzed through dual luciferase reporter assay (*n* = 3). **(C)** The enrichment of lncRNA-HRAT on Ago2 relative to IgG in H9c2 cells with or without miR-370-3p mimics was determined via RIP assay (*n* = 3). **(D)** The binding of lncRNA-HRAT and miR-370-3p in H9c2 cells with biotin-antisense or biotin-HRAT probe treatment was tested through pull-down assay (*n* = 3). **(E)** The analysis of miR-370-3p expression in H9c2 cells infected with lncRNA-HRAT or control lentivirus was conducted through qRT-PCR (*n* = 3). **(F)** The expression of miR-370-3p in the hearts from HRAT^F/F^ and HRAT CKO mice subjected to I/R (45’/3 hr) was detected by qRT-PCR (*n* = 5). **(G)** LncRNA-HRAT expression in H9c2 cells with or without miR-370-3p mimics was analyzed through qRT-PCR (*n* = 3). All the data are described as the mean ± SD. *P < 0.05, ns = not significant.

The predicted binding sites of lncRNA-HRAT and miR-370-3p are shown in Figure 5A. A dual-luciferase reporter assay was used to examine whether lncRNA-HRAT directly binds to miR-370-3p. The results indicated that miR-370-3p mimics markedly reduced luciferase activity in the WT lncRNA-HRAT reporter vector but not in the mutant lncRNA-HRAT (Figure 5B), suggesting that the binding of lncRNA-HRAT to miR-370-3p is sequence-specific. It has been reported that miRNAs bind to their targets and trigger the degradation of RNA and translational repression in an Argonaute2 (Ago2)-dependent manner (34, 35). To investigate whether lncRNA-HRAT could be regulated by miR-370-3p in this manner, an RIP assay was applied to Ago2. The results demonstrated that lncRNA-HRAT binds to miR-370-3p via Ago2 (Figure 5C). Moreover, RNA pulldown assay revealed that miR-370-3p was significantly enriched in the lncRNA-HRAT group (Figure 5D). Subsequently, the relationship between miR-370-3p and the lncRNA-HRAT was investigated. Importantly, lncRNA-HRAT overexpression significantly inhibited miR-370-3p expression (Figure 5E), whereas the expression levels of miR-370-3p were significantly increased in HRAT CKO mice subjected to I/R (Figure 5F). However, when transfected with the miR-370-3p mimic, no obvious differences were observed in the lncRNA-HRAT levels (Figure 5G). Thus, these results revealed that lncRNA-HRAT directly binds to and negatively regulates miR-370-3p.

### MiR-370-3p suppresses H/R-induced cardiomyocyte apoptosis

**FIGURE 6:**
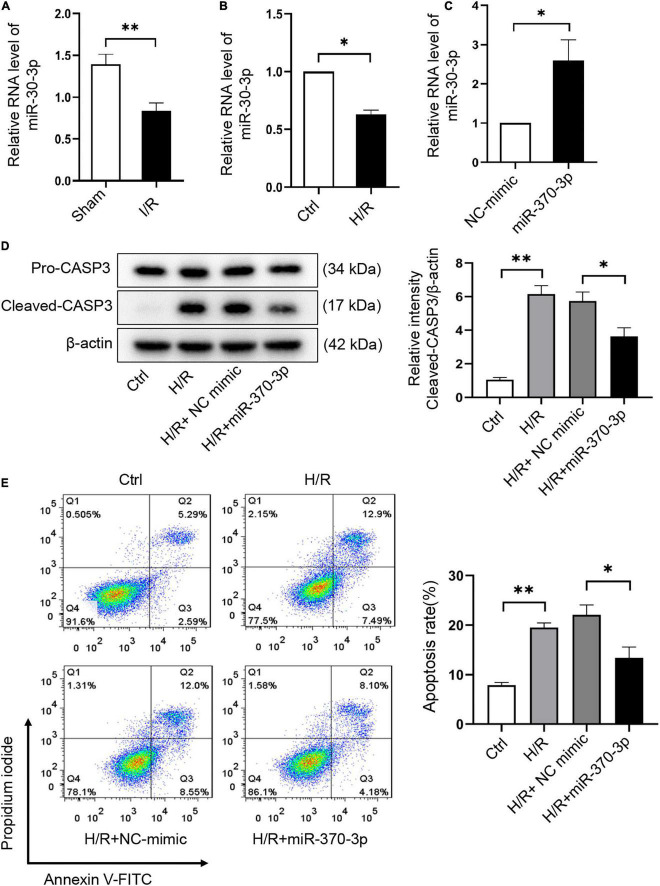
MiR-370-3p overexpression suppresses the apoptosis triggered by H/R in cardiomyocytes. **(A)** The analysis of miR-370-3p expression in I/R (45’/3 hr) and sham mice hearts was carried out through qRT-PCR (*n* = 5). **(B)** The analysis of miR-370-3p expression in Ctrl and H/R cells was employed via qRT-PCR (*n* = 3). **(C)** The analysis of miR-370-3p expression in H9c2 cells with or without miR-370-3p mimics was conducted through qRT-PCR (*n* = 3). **(D)** Protein levels of caspase-3 and cleaved-caspase-3 in different groups of cells were tested via Western blot (*n* = 3). **(E)** The apoptotic cell rate in H9c2 cells was examined via flow cytometry (*n* = 3). All the data are described as the mean ± SD. *P < 0.05, **P < 0.01.

The effect of miR-370-3p on H/R-induced cardiomyocyte injury was assessed. We revealed that miR-370-3p was markedly reduced in I/R injured mouse hearts (Figure 6A) and H/R-treated cardiomyocytes (Figure 6B). After transfection with miR-370-3p mimics, miR-370-3p exhibited a dramatic increase in expression, indicating successful transfection (Figure 6C). MiR-370-3p overexpression significantly decreased cleaved-caspase-3 expression caused by H/R, compared to that in the negative controls (Figure 6D). Moreover, a reduction in the percentage of apoptotic cells was observed when miR-370-3p was overexpressed (Figure 6E). In summary, our results demonstrated that miR-370-3p overexpression attenuates H/R-induced apoptosis in cardiomyocytes.

### RNF41 is a downstream target of miR-370-3p

**FIGURE 7:**
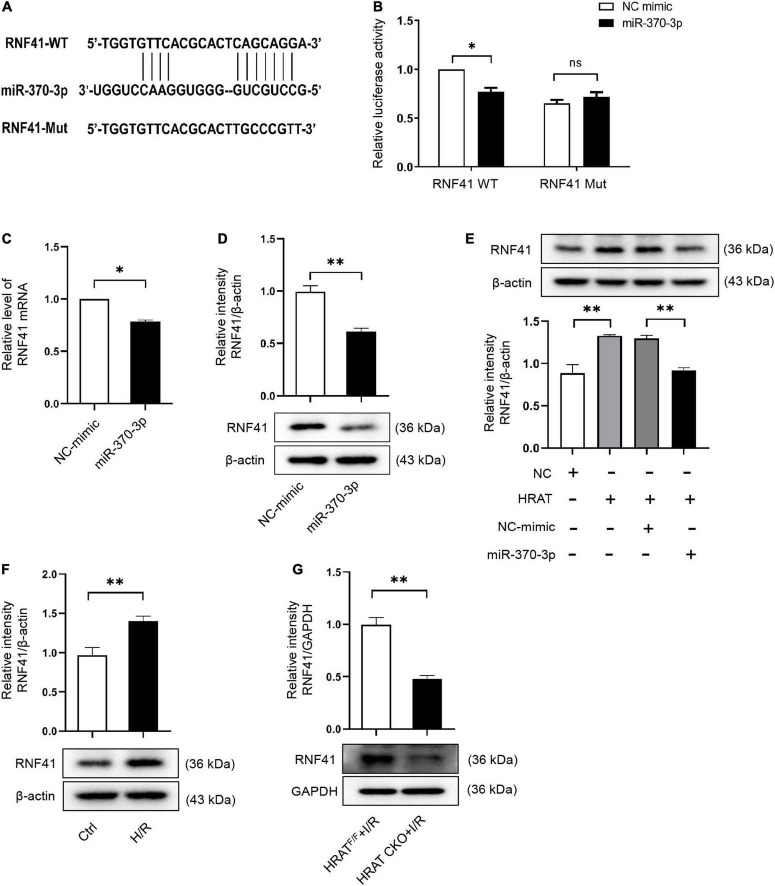
RNF41 is a downstream target of miR-370-3p. **(A)** Predicted binding site between RNF41 and miR-370-3p. **(B)** The relative luciferase activity of the RNF41 wild-type/mutant reporter in HEK-293T cells with or without miR-370-3p mimics was analyzed by dual luciferase reporter assay (*n* = 3). MRNA **(C)** and Protein **(D)** levels of RNF41 in H9c2 cells with or without miR-370-3p mimics (*n* = 3). **(E)** Protein levels of RNF41 in different groups of cells were tested via Western blot (*n* = 3). **(F)** Protein levels of RNF41 in Ctrl and H/R cells were examined by Western blot (*n* = 3). **(G)** Protein levels of RNF41 in the hearts from HRAT^F/F^ and HRAT CKO mice subjected to I/R (45’/3 hr) was detected by Western blot (*n* = 5). All the data are described as the mean ± SD. *P < 0.05, **P < 0.01, ns = not significant.

To further evaluate the mechanism underlying the contribution of the lncRNA-HRAT-miR-370-3p pathway to the pro-apoptotic effect on cardiomyocytes, we utilized TargetScan, miRWalk, and miRDB software to predict miR-370-3p target genes. We focused on RNF41 (also known as Nrdp1), which plays a pro-apoptotic role in myocardial I/R injury (36). Figure 7A displays the predicted binding sites between miR-370-3p and RNF41. The dual-luciferase reporter assay results revealed that miR-370-3p mimics significantly decreased the luciferase activity of the WT RNF41 reporter vector, but not the mutant RNF41 (Figure 7B). QRT-PCR and western blotting results indicated that miR-370-3p overexpression markedly diminished RNF41 expression (Figures 7C,D). Thus, these findings suggest that miR-370-3p directly binds to RNF41 and negatively regulates RNF41.

We further studied the impact of lncRNA-HRAT on the miR-370-3p downstream target RNF41. As shown in Figure 7E, lncRNA-HRAT overexpression increased RNF41 expression. Notably, miR-370-3p overexpression by transfection with the miR-370-3p mimic significantly diminished RNF41 expression induced by the overexpression of lncRNA-HRAT (Figure 7E). Moreover, H/R also increased the expression of RNF41 (Figure 7F). In contrast, the expression of RNF41 was significantly decreased in HRAT CKO mice subjected to I/R (Figure 7G). Therefore, these data revealed that lncRNA-HRAT serves as a ceRNA of miR-370-3p to modulate RNF41 expression.

## Discussion

Myocardial ischemia causes various conditions, such as cardiac dysfunction, arrhythmias, myocardial infarction, and even sudden death. Reperfusion causes excess cell death and an increase in infarct size, known as myocardial I/R injury (37–39). During the past few decades, many bioprocesses and protein-coding genes have been shown to participate in myocardial I/R injury. However, the mechanisms underlying myocardial I/R injury remain unclear. LncRNAs play a strong role in the development of the heart and pathophysiological processes of cardiovascular diseases (40, 41). Previous research has indicated that many lncRNAs are abnormally expressed in the heart during the early stage of reperfusion after ischemia (16). Recently, several studies have shown that lncRNAs regulate apoptosis, oxidative stress, and inflammatory responses through miRNAs or target genes, thereby modulating myocardial I/R injury (42–44). Although many lncRNAs have proved to participate in myocardial I/R injury, the existence of other lncRNAs that regulate myocardial I/R injury requires further investigation.

In this study, we identified numerous aberrantly expressed lncRNAs in H9c2 cardiomyocytes during H/R. Seven lncRNAs that may function in H/R injury were identified, and their corresponding expression was validated using qRT-PCR. The variation tendency of the two lncRNAs was consistent with the RNA sequencing results. In addition, we focused on a novel lncRNA, HRAT (lncRNA TCONS_00029632), which was significantly increased in H9c2 cardiomyocytes treated with H/R. By applying gain-of-function methods, we determined that overexpression of lncRNA-HRAT exacerbated cardiomyocyte injury by promoting apoptosis during H/R. In addition, an *in vivo* study indicated that lncRNA-HRAT expression was markedly upregulated following I/R treatment. Cardiomyocyte-specific deficiency of lncRNA-HRAT significantly decreased the release of CK in the serum, reduced myocardial infarct area, and improved cardiac dysfunction. Our results suggest that the lncRNA-HRAT has a detrimental effect on myocardial I/R injury.

Some lncRNAs influence the expression of neighboring genes by acting in cis (45). Diffusing from their transcription sites, other lncRNAs influence genes located on different chromosomes by acting in trans (11, 46, 47). In this study, we observed that lncRNA-HRAT overexpression decreased the expression of the neighboring gene Adora1, indicating that lncRNA-HRAT affects the expression of nearby genes in cis. A previous study indicated that the overexpression of Adora1 decreased I/R-induced apoptosis in the heart (48). Furthermore, in isolated rat hearts, activation of Adora1 by an agonist reduces I/R injury (49). However, the mechanisms by which lncRNA-HRAT modulates Adora1 expression in myocardial I/R injury remain unclear. This is interesting and will be investigated in future studies.

As endogenous sponge RNAs, lncRNAs may interact with miRNAs and affect miRNA target gene expression (50, 51). The interactions discovered between lncRNAs and miRNAs have inspired us (34), and we sought to identify miRNAs accompanied by complementary base pairing with lncRNA-HRAT. Both luciferase and Ago2 RIP assays demonstrated that miR-370-3p binds to lncRNA-HRAT directly. RNA pulldown assay further verified the directional binding of lncRNA-HRAT to miR-370-3p. In addition, lncRNA-HRAT overexpression declined the expression of miR-370-3p in H9c2 cardiomyocytes. However, miR-370-3p overexpression showed no obvious differences in the lncRNA-HRAT levels. Our studies revealed that lncRNA-HRAT binds to miR-370-3p directly and negatively regulates its expression of miR-370-3p.

MiR-370-3p is involved in suppressing the proliferation and invasion in various types of cancer cells (21–23). Hou et al. reported that miR-370-3p also suppresses the proliferation of human vascular smooth muscle cells in cerebral aneurysms by targeting KDR and blocking the AKT/FOXO1 signaling pathway (52). Recent studies have suggested that miR-370-3p inhibits hypoxia-induced cardiomyocyte injury (24–26). In this study, we revealed that miR-370-3p overexpression inhibited H/R-induced apoptosis in cardiomyocytes.

RNF41 (also known as Nrdp1), a RING finger E3 ubiquitin ligase, is mainly expressed in skeletal muscle, brain, and heart (36). RNF41 regulates oxidative stress, inflammation, apoptosis, and cell proliferation (53–55). A previous study demonstrated that overexpression of RNF41 promotes cardiomyocyte apoptosis and inflammation induced by myocardial I/R injury (36). However, the relationship between miR-370-3p and RNF41 in I/R injury remains unknown. In this study, we found that RNF41 is a downstream target of miR-370-3p in I/R injury. MiR-370-3p negatively regulates RNF41 expression.

LncRNA-H19 binds to miR-370-3p directly and serves as a ceRNA to facilitate the epithelial-mesenchymal transition (EMT) in ovarian cancer cells induced by TGF-β (56). Recent studies indicate that lncRNA-FGF14-AS2 serves as a tumor inhibitor by sponging miR-370-3p, which has an inhibitory effect on FGF14, thereby restraining the migration and invasion of breast cancer cells (57). Similarly, our results revealed that lncRNA-HRAT serves as a ceRNA of miR-370-3p and increases RNF41 expression, thereby aggravating H/R-induced apoptosis in cardiomyocytes.

In summary, we identified and characterized a novel lncRNA HRAT, which regulates cardiomyocyte apoptosis and myocardial injury during I/R. Our research revealed that the lncRNA-HRAT/miR-370-3p/RNF41 pathway plays a strong role in regulating cardiomyocyte apoptosis and myocardial injury. Our findings imply that lncRNA-HRAT may be a possible target for the treatment of myocardial I/R injury. This study provides novel insights into the mechanisms underlying myocardial I/R injury.

## Data availability statement

The datasets presented in this study can be found in online repositories. The names of the repository/repositories and accession number(s) can be found below: https://figshare.com/s/dc534de95cfb6cb3378b.

## Ethics statement

The animal study was reviewed and approved by Animal Research Committee of Center of Central South University (grants 2019sydw0160).

## Author contributions

XZ, DS, and DY conceived and designed this study and wrote the manuscript. XZ, TZ, FY, JD, YT, and YL conducted the experiments. XZ and ML performed data analysis. FY interpreted the results of the experiments. All authors reviewed and approved the final version of the manuscript.

## Abbreviations

I/R: ischemia/reperfusion
lncRNA: long non-coding RNA
H/R: hypoxia/reoxygenation
HRAT: hypoxia/reoxygenation associated transcript
CASP3: caspase-3
miRNA –: microRNA
FISH: fluorescence *in situ* hybridization
RNF41: ring finger protein 41
TTC: 2, 3, 5-triphenyl tetrazolium chloride
CK: Creatine kinase
RIP: RNA immunoprecipitation.
