# Supplementary material for: Deficiency of a novel lncRNA-HRAT protects against myocardial ischemia reperfusion injury by targeting miR-370-3p/RNF41 pathway
Source: Front Cardiovasc Med. 2022 Sep 12;9:951463. doi: 10.3389/fcvm.2022.951463 (PMC9510651; doi:10.3389/fcvm.2022.951463)
Supplement: Supplementary file 1 [file Table_1.docx]

**Figure. S1. Rat HRAT transcript sequence**

CAGGTATTAACTCCTATTCCTAAAAGCTACTCATCCGACCTGGTTGCTCGTGCTGGTGACACAGGAATCTTTTGATGGCTAGATGTCTTCATTTTCCCCTCCCCAGTACCCAGGGTTTCATCGTCACGCAAGGCAAGCCCTCTACCATTGAGTTATTTCGCTCAGCCCAAAGTTAGACGACCTCATCACACAGGACCGGCGCCTGGAGCACAATAGTGTGGGGTAGACGGTCCAGAGGAGGTTCTACAAGACAGCGGGACCATGTTATCTTTGCGGGCTCTTTTGCCCCTCGTGGCATCGCGGGTGACATGACAATTATTTTTTCTCCCTTTTTGCAGCCTACAGAGGCTGAGGGAGGGCAGAGAGACTGCGGAGCGGTGTTTGGCGCTCTTGTCTGGAGCTCGGGGTCCCCCTGCTGGAGGCACTGGAACCTGGGCGGGGCTTTAAGACGAAGGGCAGACACCCTCAGGTGATAACTGTGGCAAACTCAGGATGAGGCGGAGCTGCTGGAACTCAGCTGGAAGGCCCAAGGGACCAGCCCTAGGAATGTTAGACAAATACGCCACCCGGATGGTGACTCTGGTCTCCTGATGCCCAGTCTAAATCCTTTGTTCCCAGCCCCGCTACTAATTATGTTTTCAAGCAAAATCTCCTGAAACTGCCCAAGGAGCTGGGAGCGCTCCCTGCATTGCGGAGATCTCGGAAGATATGGTTCCCAGCACCCATGCTTCCTGCCAGCCCGCCTCAGGAATCTTTCAGCACAGAGCTTTCGCCTCCAGTACTTCTGGGAACACCACGTATCTGGGCACAGAACCCTTAGGTCACCCAATTCTCCTCAAAGGCTGTGCATACTTGGTCTCTAGGGCCTAGTAAATTCAGCTCAAATTTCCCTACTGGGAATGTGACCAAGAAGCCTCAGTTTTGCCGTCTGTAAAATGCAAACTGTAGAATTTTCTGAATGAGCAGCTGGGAGAATTTGGTGGTACTGTGAGGTTGGTCCCCCTTGAGCATTGACACACGTCCCTAAGTATTGATGTTTGCACGGTTCTTTGGCCCTTCCCACCCTAACATGAAACTAACAAGAAACTGGGTAACTACAGCAGACAGGTAGGAGTTGTCCATGGAGAAGTATGGCAGGGCCCTGCCACAGGTTTGAGATTGTCCCAACCTAGCCACCTCCTCAGTCTTCCACCCAGTCGACAGCAGGAGTGGCAGCTGCTTCCCTCTGCTGCCCAATCTCTGAGGCCTGGCACAGCTGCTGGGGTGGCCCAAATGCCTCAGGTCTAGGACTACACAAAG

**Figure. S2. Mouse HRAT transcript sequence**

CAGAAATTAACTCCTATTCCTAAAAGCCAGTCACTCGACCTGGTCGCTCGTGCTAGGGACAGAGGGGTCTTTCGATGTCTAGACGTCTTCATTTTCCCCTCCCCAGTACCCAGGGTGTCACTTGCAAGGCAAGCCCTCCACCACTGAGCTGTTTCGATCACCCCAAAGTTGGACAACCTCATCACACAGGGCCGGCGCGGCTCCCGGAGCAGACGGTCCAGAATGAGATTCAAGACAGCAGAACCACGTTATCTTTGTGGGTTCTTTAGCCCCTCGTGGCATCGCGGGTGACATGGCAATAATTCTCCCTTTTTGCAGCCTACAGAGGCTGAGGGAGGGCAGAGAGGCTACAGATCAACGTTCAGTGCCCTTGTCTGGAGCTCGGGGTCCCCCTGCTGGCGCACTGGAGCCTGGGCGGGGCTTTGAGCCGAAGGGCGGACACAATCAGGTGACAACTCTGGGGAACGTGGCGAATGAGGTGGAGCTGCTGGAACTCAGCTGGAAGGCCCAGGGGACCAGCCCTAGGAATGTCCAACAAATATGCCACCCCGATGGCGACTTTGGTCTCCTGGTGCCCAATCCAAATCCTTTGTTACCAGCCCCGCTGCTTCTTATGTTTGCAGGCAAAATCTCCTGAACATGCCCGAAGAGCATGTGTTGGGAGCCCTCCCTGCTCTGGGGAAATCTTCAAAGACGGGGTTCCCAGCACCGTCTGCTTCCGGCCAGCCTGCCTCAGGAGTGGTTCATCGCAGACCTTCCACCTCCAGTACTTCTGGGAACACCATGCGTCTGGGCACAGAACCCTAGGTCACCCAATTCTCCTCAAAGGCCTCCAGGGCCTAGTAAATTTGGCTCAAATGTCCCTAACTGGGAATGTGACCAGGAAGCCTCAGTTTTGCCGTCTATAAAATGCAAACTATCGACTTTTCTATGTGTGTGGCTGGGAGGATTTGGTGGTATTGTGAAGTTGGTCGCACTTGAGCATTTGACACACACCCCTAAGCACTGATGTTTGCATAGCTTTTTGGCCCTTCCCACCCTAACATGTAACTAATGGCAGGTAGGAGTTGTCCACGAGGAGTAGGGCACCGCCCTGCCACGTGTTTGAGATTTTCCCCACCTAGCCAACTCCTTGCTCTTCCACCCAGTCTACAACAGGAGGGGTGGCAGCTGTTTCCCTCGGCTGCCCAGTCTCTGACCCAGCCAAAGGCTGGCACAGCTGCTGGGGTGGCCCAAATGCTACACAAAGGACAGCTGGGGCTGAAGGAGGACAAAGATG


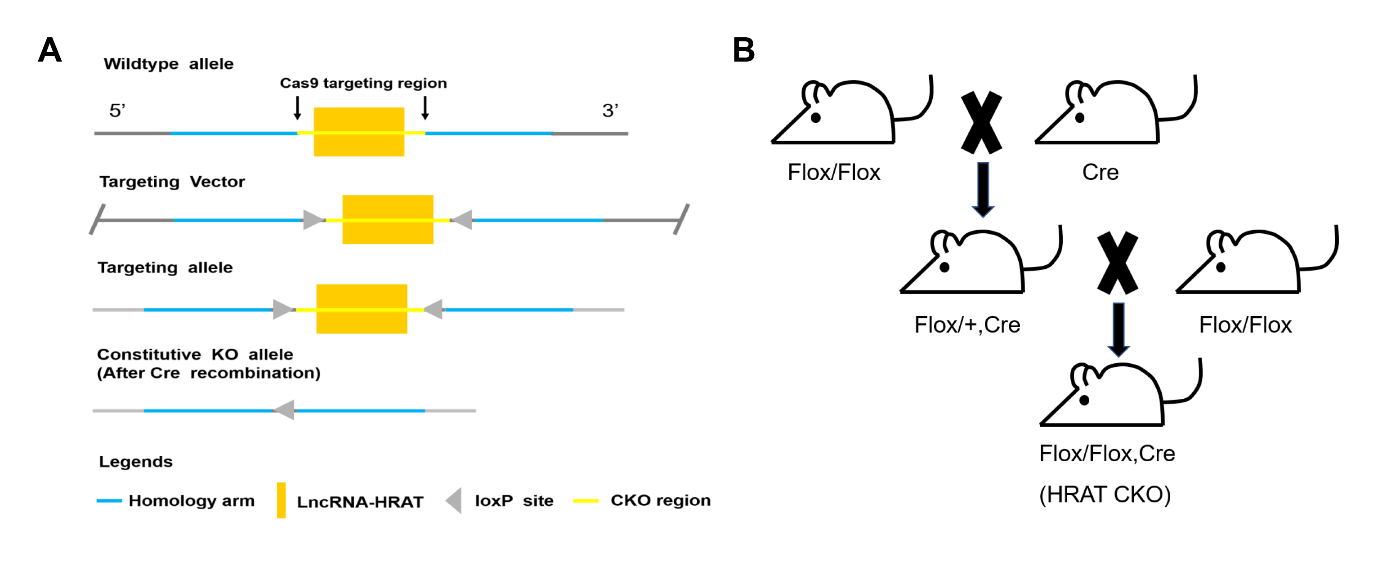


**Figure. S3.** The strategy for generating HRAT CKO mice. **(A)** Strategy for creating HRAT conditional mice using Cre-LoxP recombinant system. **(B)** HRAT floxed mice were crossed with α-MyHC-Cre transgenic mice to generate HRAT CKO mice.


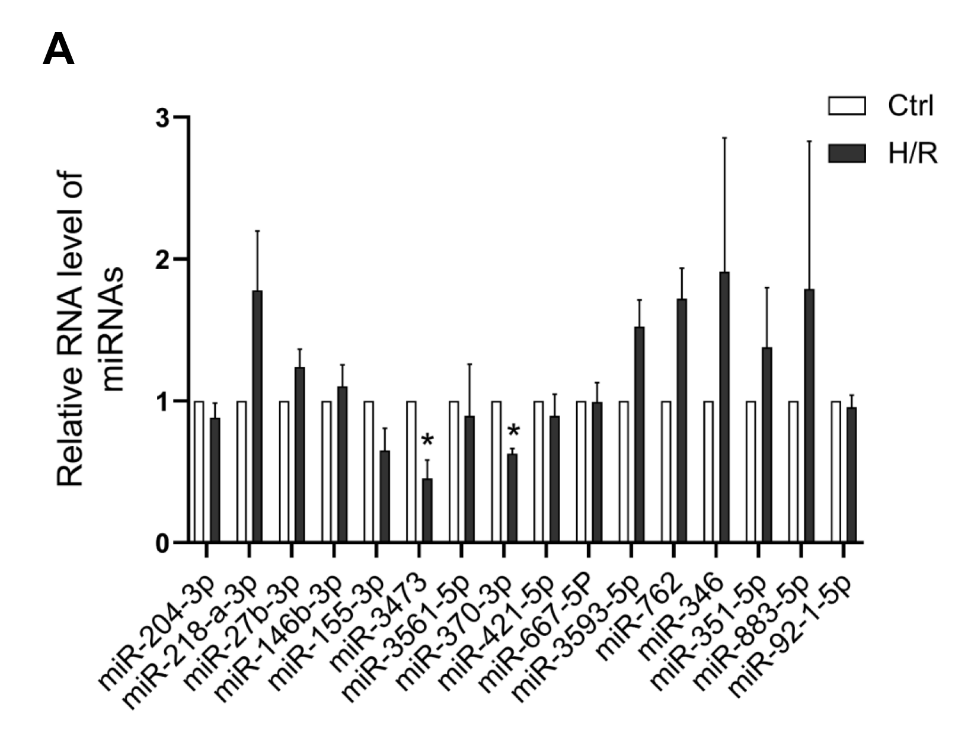


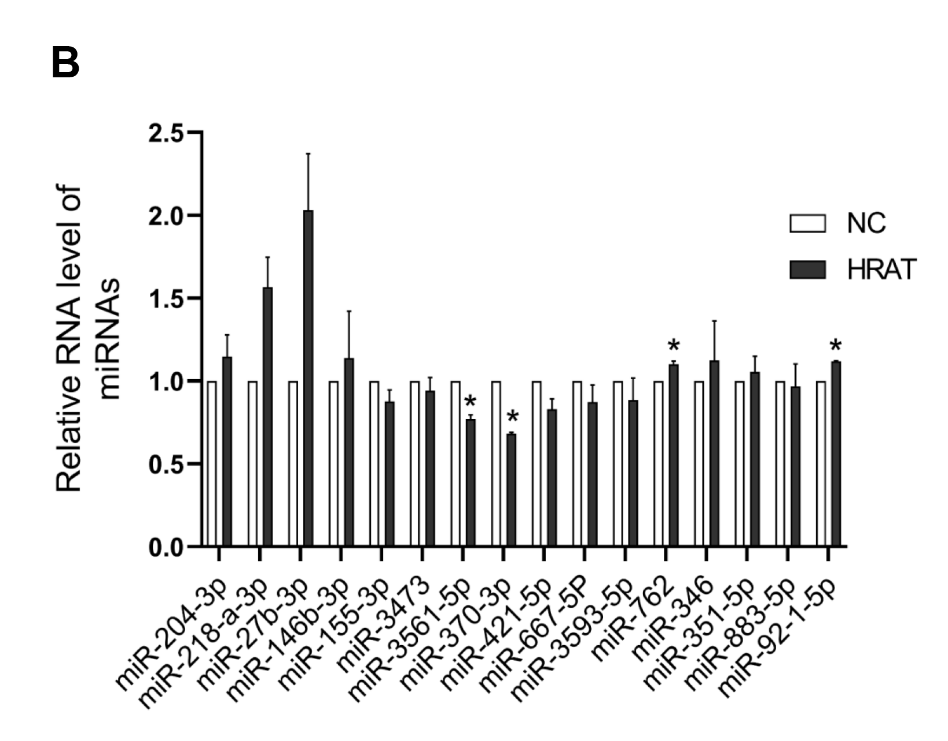


**Figure. S4.** The screening of HRAT potential interacting miRNAs. **(A)** The expression of miRNAs were analyzed by qRT-PCR in H/R group and Ctrl Group cells (n=3). **P*<0.05 vs Ctrl. **(B)** The expression of miRNAs in H9c2 cells infected with lncRNA-HRAT lentivirus or the control lentivirus were analyzed by qRT-PCR (n=3). **P*<0.05 vs NC.

**Supplemental Table 1**. Primers sequence used in this study.

| PCR Primers |  |
| --- | --- |
| Gene | sequence |
| Rat-TCONS_00029632-F | 5'-GGGTTTCATCGTCACGCAAG-3' |
| Rat-TCONS_00029632-R | 5'-GGACCGTCTACCCCACACTA-3' |
| Rat-TCONS_00029844-F | 5'-TTTGCGACCAGAGGAACCAT-3' |
| Rat-TCONS_00029844-R | 5'-CTCTCGCTAGAACCACAGCC-3' |
| Rat-TCONS_00030327-F | 5'-GCTGAGTCCAAGACACGCTA-3' |
| Rat-TCONS_00030327-R | 5'-GGATGGTGCTGTGGTTAGGT-3' |
| Rat-TCONS_00033981-F | 5'-GTCTGGGGAATGTGTTGGCT-3' |
| Rat-TCONS_00033981-R | 5'-AACCTCTGGGTAAGCTTGGTC-3' |
| Rat-TCONS_00078210-F | 5'-ATTGCTGCCAGAGCCAGAT-3' |
| Rat-TCONS_00078210-R | 5'-TGGAACGCACGGACATCA-3' |
| Rat-TCONS_00100522-F | 5'-AGAAGCTTGGTTGCATTGCG-3' |
| Rat-TCONS_00100522-R | 5'-CCCGCTCTGAATAGCGACAA-3' |
| Rat-TCONS_00078211-F | 5'-TTTCTCGGGATCCTCTCCGT-3' |
| Rat-TCONS_00078211-R | 5'-CCCACACATTCCACAACCGA-3' |
| Rat-GAPDH-F | 5'-ACAGCAACAGGGTGGTGGAC -3' |
| Rat-GAPDH-R | 5'-TTTGAGGGTGCAGCGAACTT -3' |
| Rat-Adora1-F | 5'-GCCTTCCAGGCTGCCTACAT-3' |
| Rat-Adora1-R | 5'-AGTGCCTGGTTCACCTTCACA-3' |
| Rat-miR-370-3p | 5'-GCCTGCTGGGGTGGAACCTG-3' |
| Rat-miR-204-3p | 5'-CTGGGAAGGCAAAGGGACGTT-3' |
| Rat-miR-346 | 5'-TGTCTGCCTGAGTGCCTGCCT-3' |
| Rat-miR-351-5p | 5'-GGAGCCCTTTGAGCCTGAAAA-3' |
| Rat- miR-883-5p | 5'-CGGGGCTGAGAGAAGTAGCA-3' |
| Rat- miR-92a-1-5p | 5'-GGGAGGTTGGGATTTGTCGC-3' |
| Rat- miR-146b-3p | 5'-GCGGGACTCAGTTCTGGTGAAAA-3' |
| Rat- miR-155-3p | 5'-CCGCCCTCCTACCTGTTAGC-3' |
| Rat-miR-3473 | 5'-CTAGGGCTGGAGAGATGGCTAA-3' |
| Rat-miR-3561-5p | 5'-CGGGTGTCAATCCAGGGTAGA-3' |
| Rat-miR-421-5p | 5'-GGGGGGCCTCATTAAATGTTTGTT-3' |
| Rat-miR-667-5p | 5'-GGTGCTGGTGGAGCAGTGAG-3' |
| Rat-miR-762 | 5'-GGGCTAGGGCCGGGAAAAAA-3' |
| Rat-miR-27b-3p | 5'-CACAGTGGCTAAGTTCTGCAAAAA-3' |
| Rat-miR-218a-1-3p | 5'-CATGGTTCCGTCAAGCACAAAAA-3' |
| Rat-miR-3593-3p | 5'-CAACCTTAAGGGGGCCTCAAAAA-3' |
| Mouse--miR-370-3p | 5'-TATCGCCTGCTGGGGTGGAA-3' |
| Mouse -TCONS_00029632-F | 5'-GTTATCTTTGTGGGTTCTTTAGCC-3' |
| Mouse -TCONS_00029632-R | 5'-GTTGATCTGTAGCCTCTCTGCC-3' |
| Mouse -GAPDH-F | 5'-GCGACTTCAACAGCAACTCC -3' |
| Mouse -GAPDH-R | 5'-CACCCTGTTGCTGTAGCCGTA -3' |

**Supplemental Table 2.** The potential interacting miRNAs of lncRNA-HRAT was analyzed by using miRanda, pita and RNAhybrid softwares.

| lncRNA | miRNA | miRanda | pita | RNAhybrid |
| --- | --- | --- | --- | --- |
| lncRNA-HRAT | Rat -miR-346 | 1 | 1 | 1 |
| lncRNA-HRAT | Rat-miR-351-5p | 1 | 1 | 1 |
| lncRNA-HRAT | Rat-miR-27b-3p | 1 | 1 | 1 |
| lncRNA-HRAT | Ra -miR-92a-1-5p | 1 | 1 | 1 |
| lncRNA-HRAT | Rat-miR-204-3p | 1 | 1 | 1 |
| lncRNA-HRAT | Rat-miR-218a-1-3p | 1 | 1 | 1 |
| lncRNA-HRAT | Rat -miR-421-5p | 1 | 1 | 1 |
| lncRNA-HRAT | Rat-miR-370-3p | 1 | 1 | 1 |
| lncRNA-HRAT | Rat-miR-883-5p | 1 | 1 | 1 |
| lncRNA-HRAT | Rat-miR-146b-3p | 1 | 1 | 1 |
| lncRNA-HRAT | Rat -miR-667-5p | 1 | 1 | 1 |
| lncRNA-HRAT | Rat-miR-3561-5p | 1 | 1 | 1 |
| lncRNA-HRAT | Rat-miR-3593-5p | 1 | 1 | 1 |
| lncRNA-HRAT | Rat-miR-3473 | 1 | 1 | 1 |
| lncRNA-HRAT | Rat-miR-155-3p | 1 | 1 | 1 |
| lncRNA-HRAT | Rat-miR-762 | 1 | 1 | 1 |

Note: “1” represents the binding of miRNA to lncRNA-HRAT
