# Supplementary material for: Deficiency of a novel lncRNA-HRAT protects against myocardial ischemia reperfusion injury by targeting miR-370-3p/RNF41 pathway
Source: Front Cardiovasc Med. 2022 Sep 12;9:951463. doi: 10.3389/fcvm.2022.951463 (PMC9510651; doi:10.3389/fcvm.2022.951463)
Supplement: Supplementary file 3 [file Data_Sheet_1.ZIP › Original Source Data╫ε╨┬░μ/Figure 2/Figure 2B.pptx]

## Slide 1
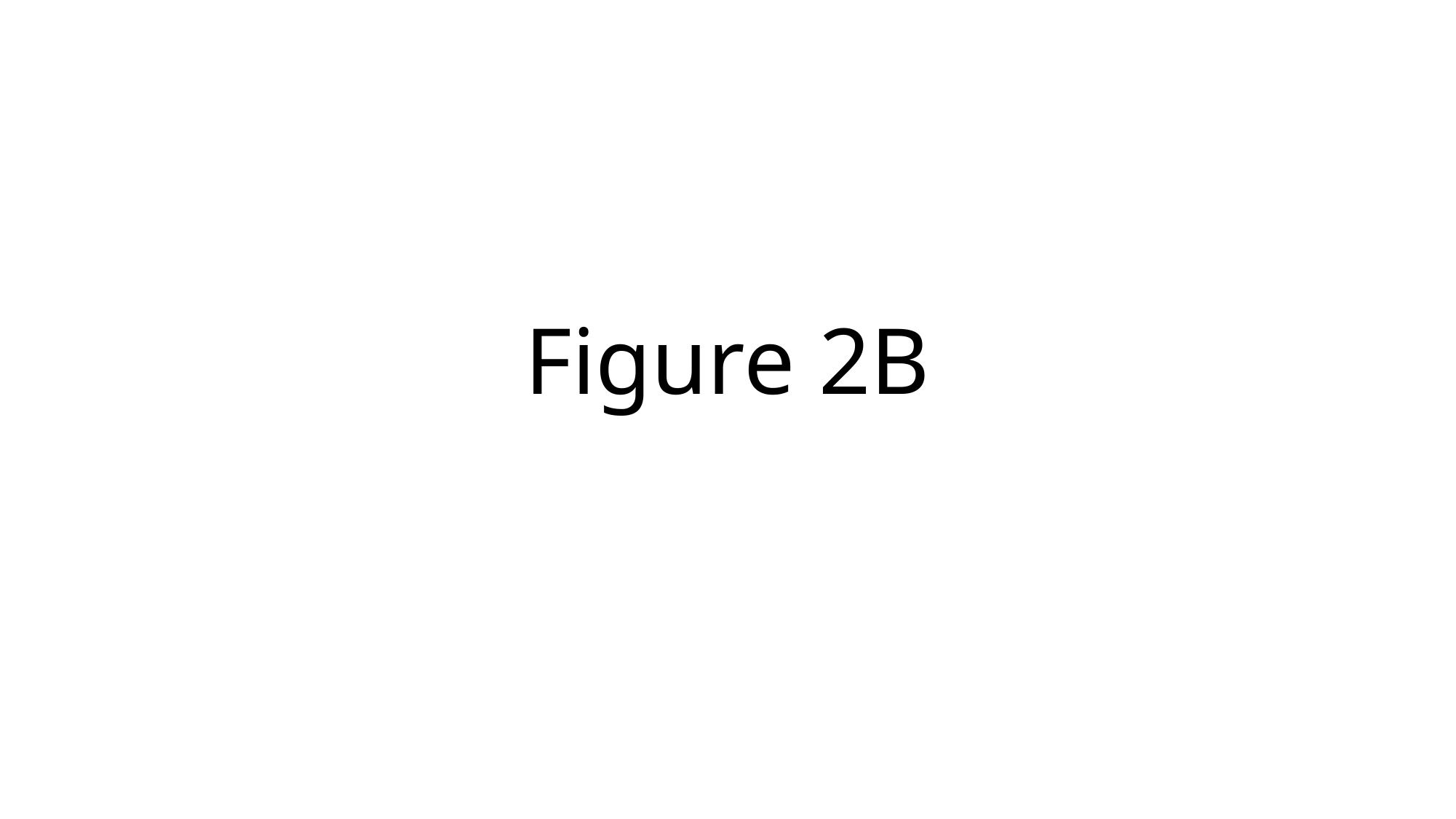

# Figure 2B

## Slide 2
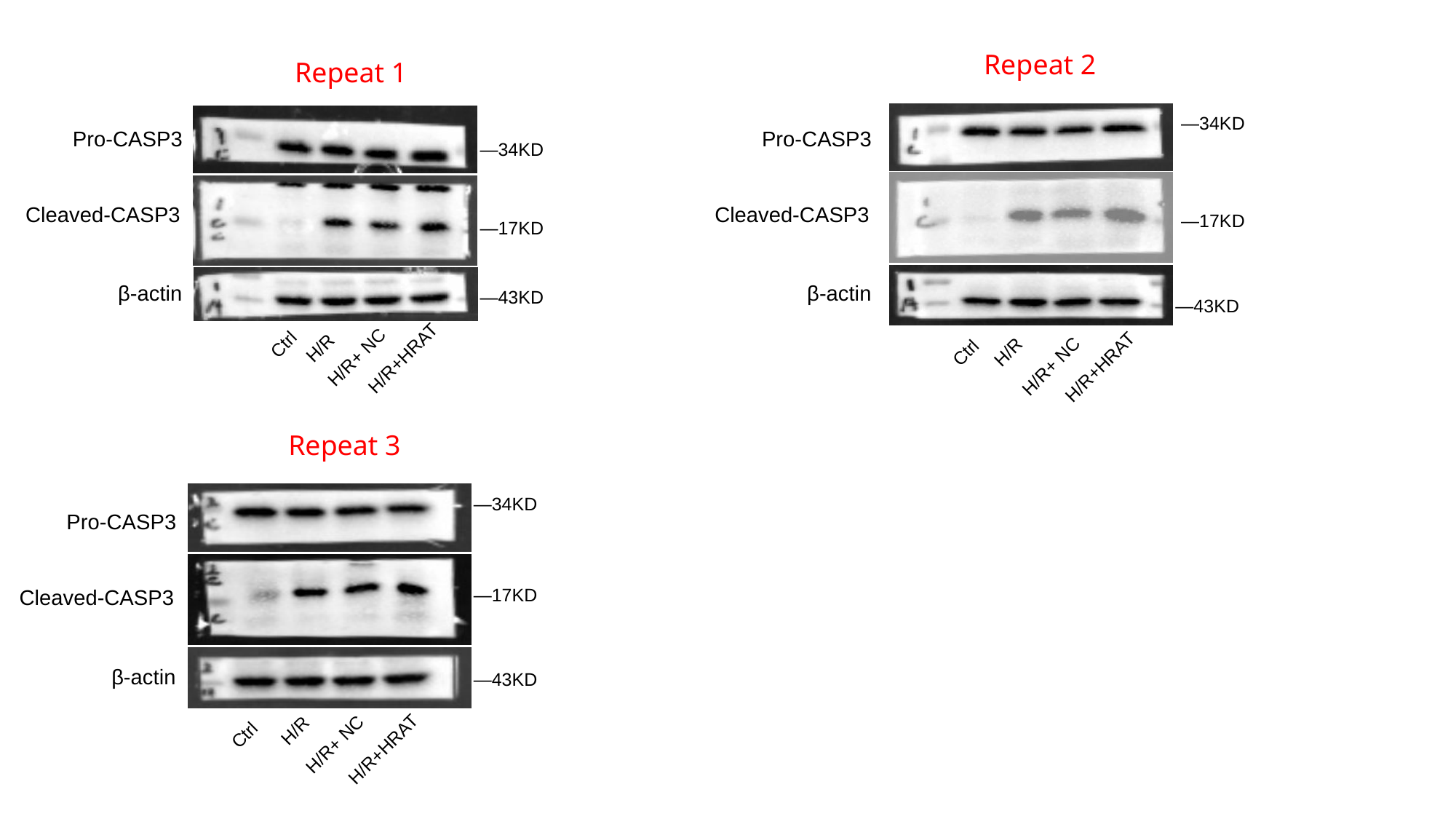

Repeat 2
Repeat 1
—34KD
Pro-CASP3
Pro-CASP3
—34KD
Cleaved-CASP3
Cleaved-CASP3
—17KD
—17KD
β-actin
β-actin
—43KD
—43KD
Ctrl
Ctrl
H/R
H/R
H/R+ NC
H/R+HRAT
H/R+ NC
H/R+HRAT
Repeat 3
—34KD
Pro-CASP3
—17KD
Cleaved-CASP3
β-actin
—43KD
Ctrl
H/R
H/R+ NC
H/R+HRAT
