# Supplementary material for: Deficiency of a novel lncRNA-HRAT protects against myocardial ischemia reperfusion injury by targeting miR-370-3p/RNF41 pathway
Source: Front Cardiovasc Med. 2022 Sep 12;9:951463. doi: 10.3389/fcvm.2022.951463 (PMC9510651; doi:10.3389/fcvm.2022.951463)
Supplement: Supplementary file 3 [file Data_Sheet_1.ZIP › Original Source Data╫ε╨┬░μ/Figure 2/Figure 2E.docx]

**1:**


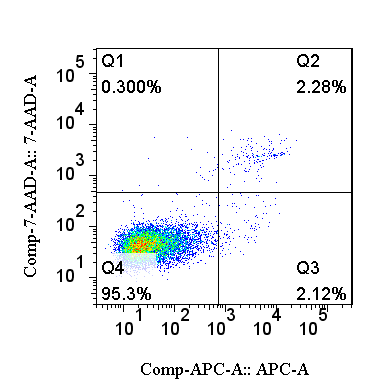

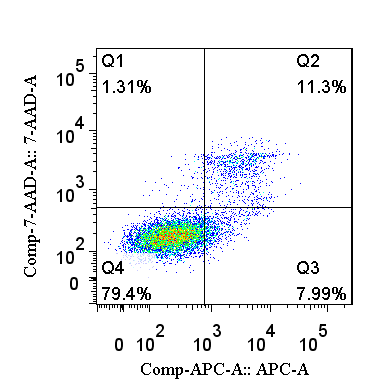

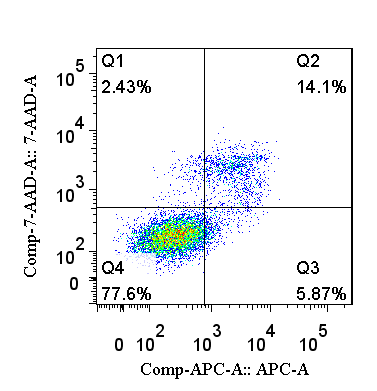

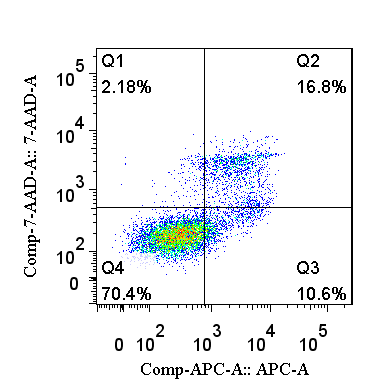


H/R+HRAT

H/R+NC

H/R

Ctrl

**2：**


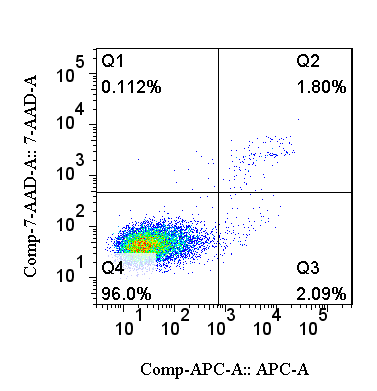

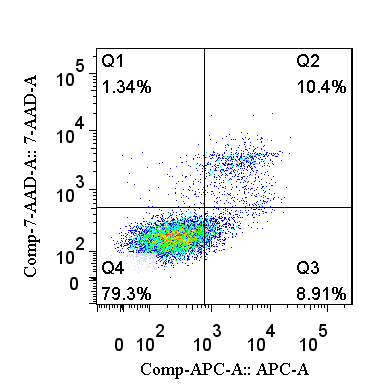

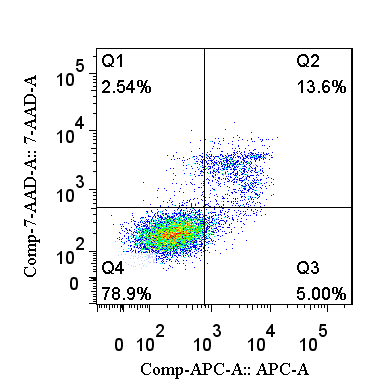

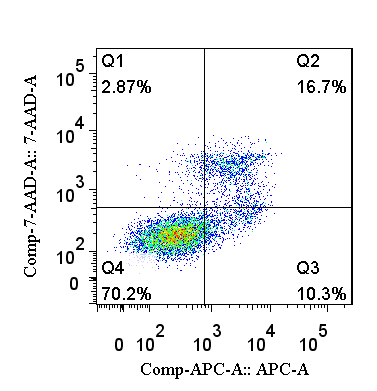


H/R+9632

H/R+NC

Ctrl

H/R

**3:**


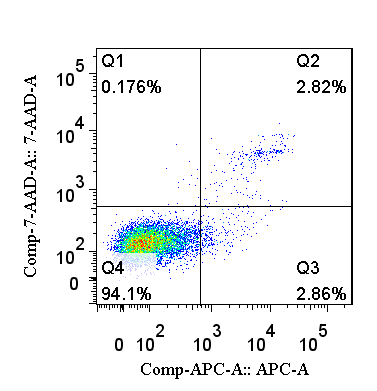

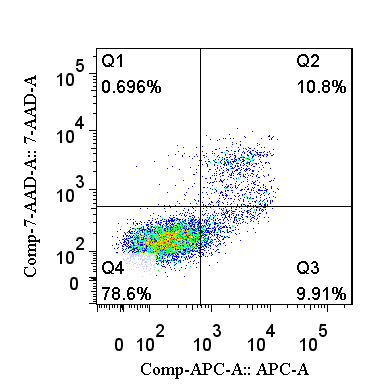

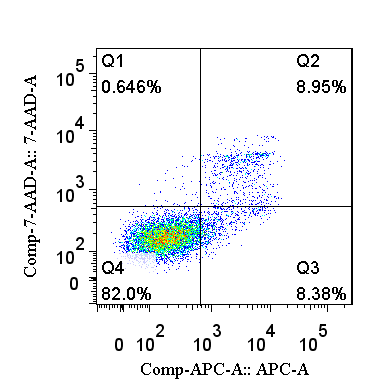

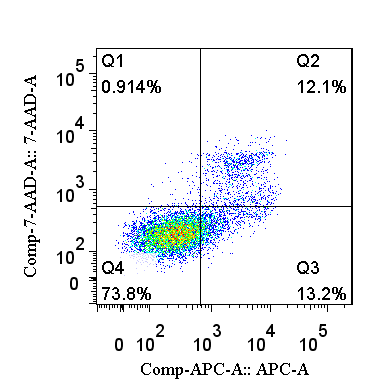


H/R+HRAT

H/R+NC

H/R

Ctrl

**Apoptosis rate (%)**

|  | Ctrl | H/R | H/R+NC | H/R+HRAT |
| --- | --- | --- | --- | --- |
| Repeat 1 | 4.4 | 19.29 | 19.97 | 27.4 |
| Repeat 2 | 3.89 | 19.31 | 18.6 | 27 |
| Repeat 3 | 5.68 | 20.71 | 17.33 | 25.3 |
