# Supplementary material for: Deficiency of a novel lncRNA-HRAT protects against myocardial ischemia reperfusion injury by targeting miR-370-3p/RNF41 pathway
Source: Front Cardiovasc Med. 2022 Sep 12;9:951463. doi: 10.3389/fcvm.2022.951463 (PMC9510651; doi:10.3389/fcvm.2022.951463)
Supplement: Supplementary file 3 [file Data_Sheet_1.ZIP › Original Source Data╫ε╨┬░μ/Figure 6/Figure 6E.docx]

**1:**


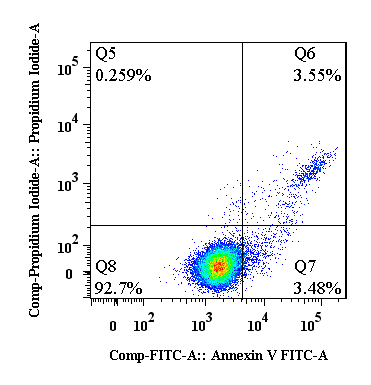

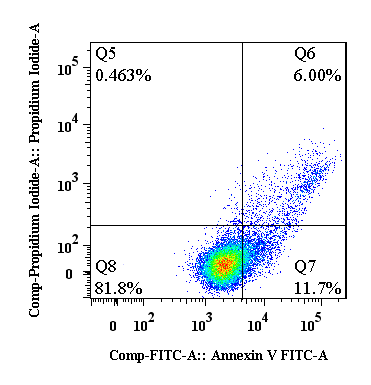

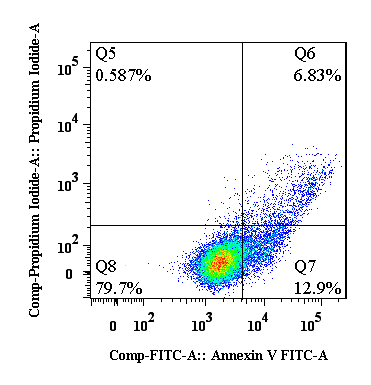

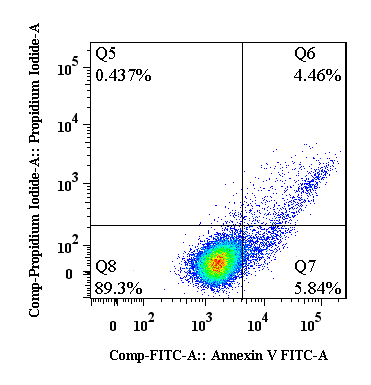


H/R+miR-370-3p

H/R+NC mimic

H/R

Ctrl

**2:**


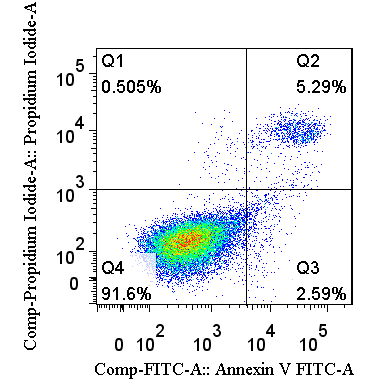

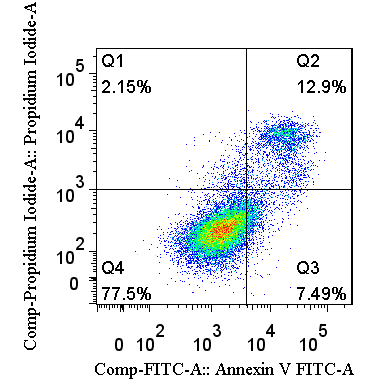

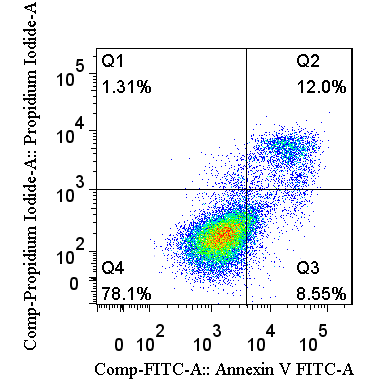

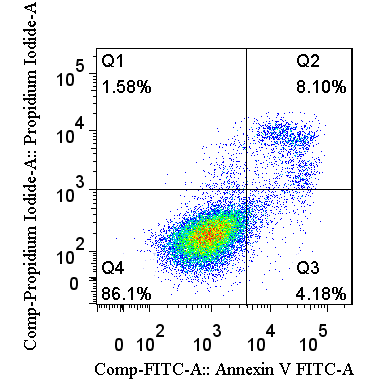


H/R+miR-370-3p

H/R+NC mimic

H/R

Ctrl

**3:**

**
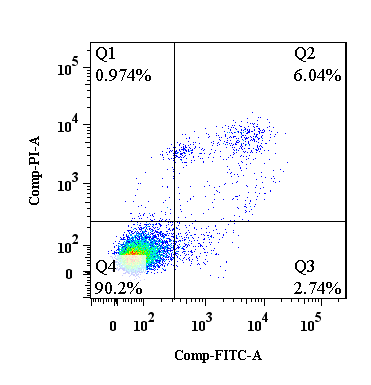

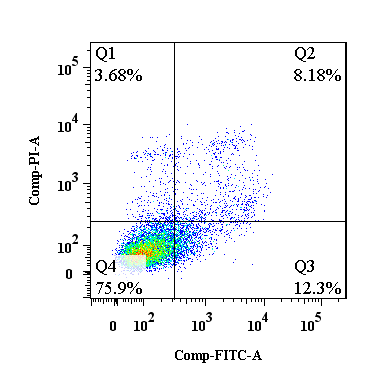

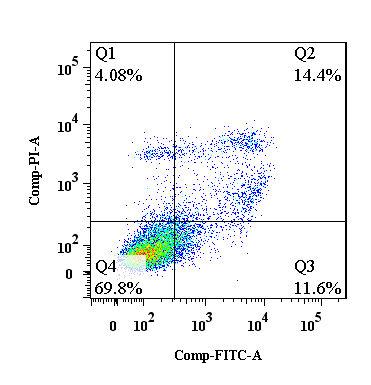

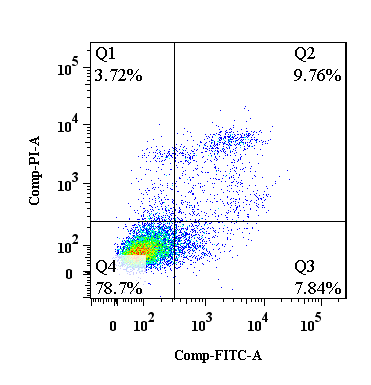
**

H/R+NC mimic

H/R+miR-370-3p

H/R

Ctrl

**Apoptosis rate (%)**

|  | Ctrl | H/R | H/R+NC-mimic | H/R+miR-370-3p |
| --- | --- | --- | --- | --- |
| Repeat 1 | 7.03 | 17.7 | 19.73 | 10.3 |
| Repeat 2 | 7.88 | 20.39 | 20.55 | 12.28 |
| Repeat 3 | 8.78 | 20.48 | 26 | 17.6 |
