# Supplementary material for: Deficiency of a novel lncRNA-HRAT protects against myocardial ischemia reperfusion injury by targeting miR-370-3p/RNF41 pathway
Source: Front Cardiovasc Med. 2022 Sep 12;9:951463. doi: 10.3389/fcvm.2022.951463 (PMC9510651; doi:10.3389/fcvm.2022.951463)
Supplement: Supplementary file 3 [file Data_Sheet_1.ZIP › Original Source Data╫ε╨┬░μ/Figure 7/Figure 7E.pptx]

## Slide 1
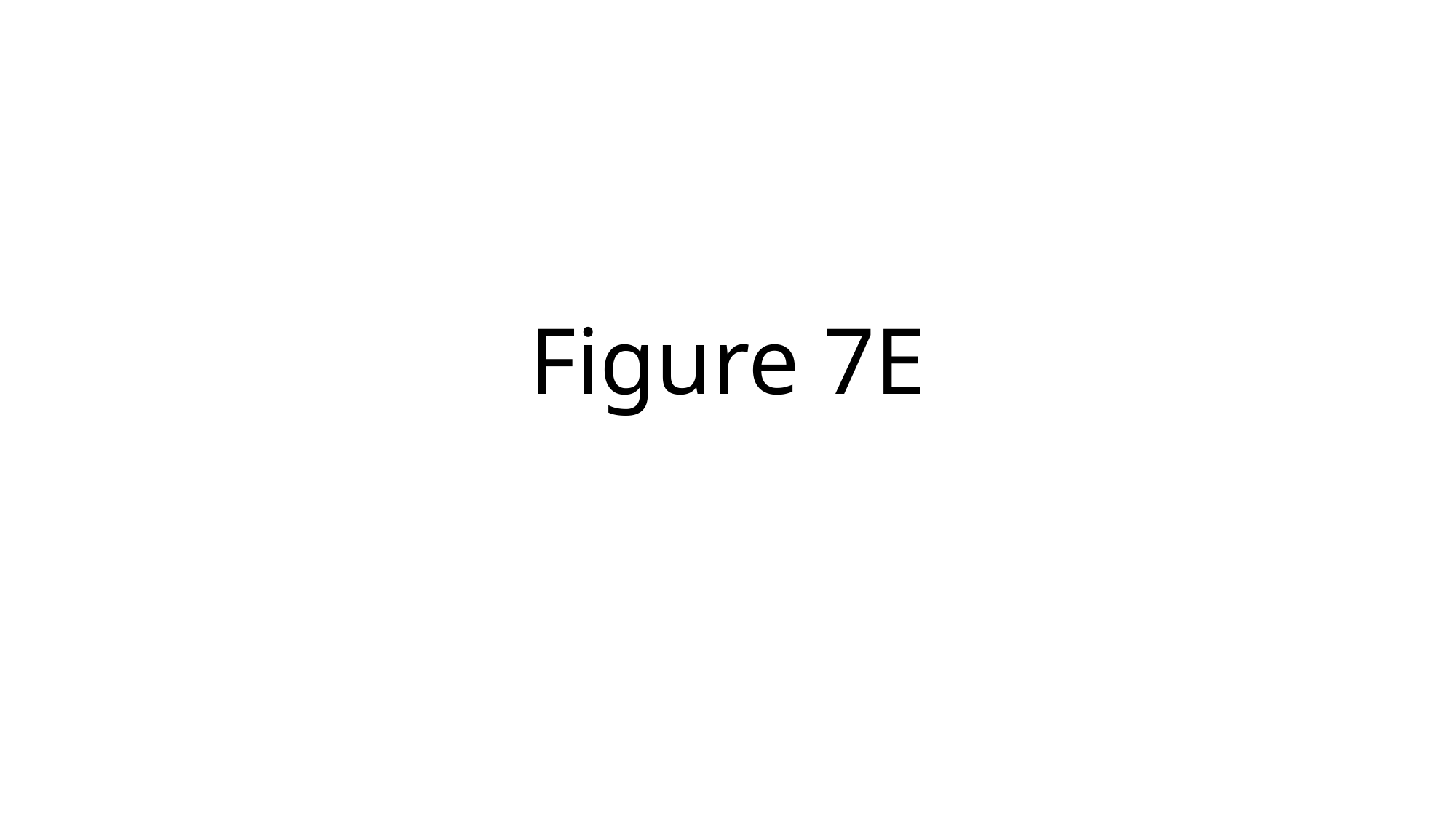

# Figure 7E

## Slide 2
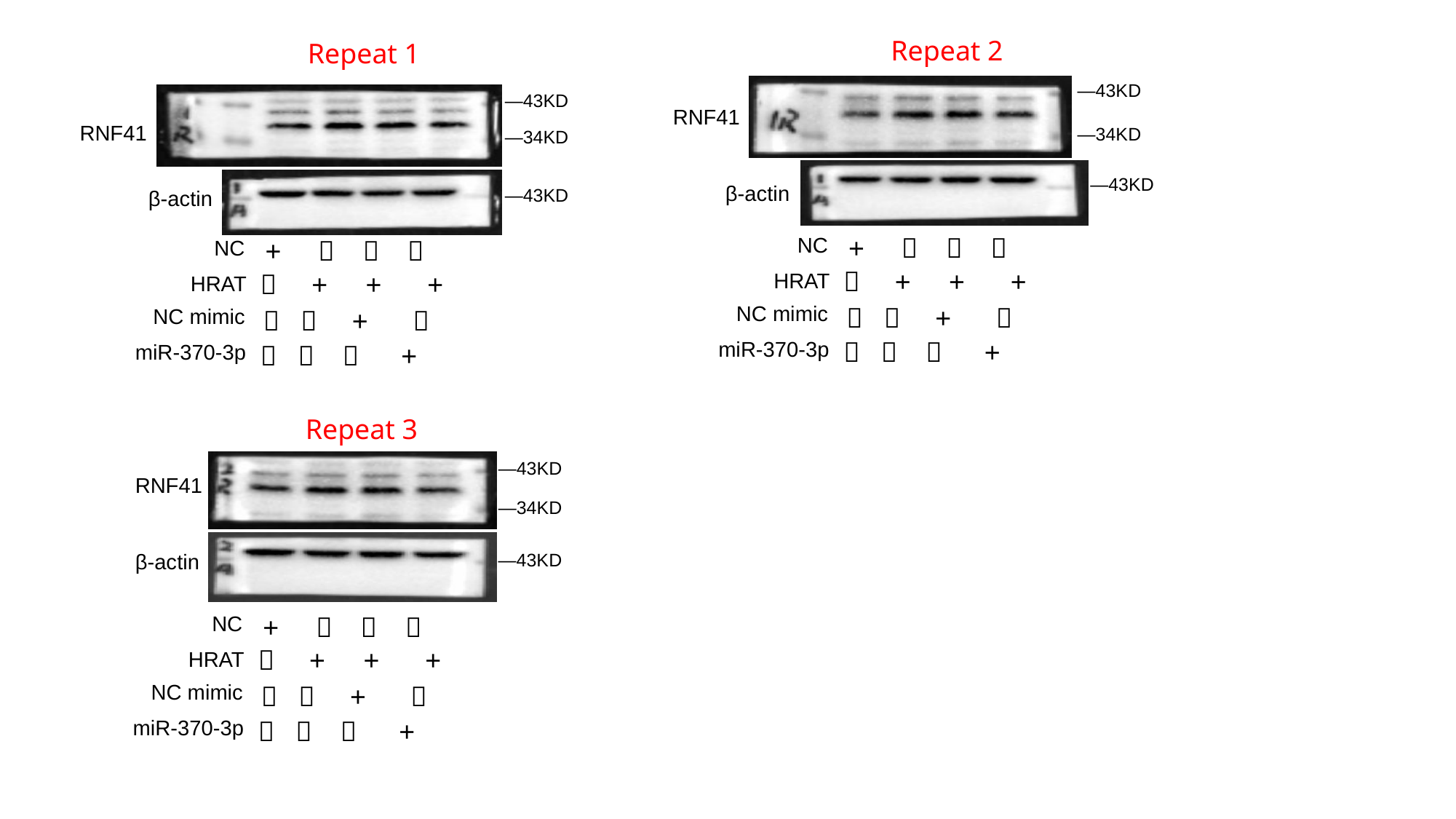

Repeat 2
Repeat 1
—43KD
—43KD
RNF41
RNF41
—34KD
—34KD
—43KD
β-actin
—43KD
β-actin
 + － － －
 + － － －
NC
NC
－ + + +
HRAT
－ + + +
HRAT
NC mimic
－ － + －
NC mimic
－ － + －
－ － － +
miR-370-3p
－ － － +
miR-370-3p
Repeat 3
—43KD
RNF41
—34KD
—43KD
β-actin
 + － － －
NC
－ + + +
HRAT
NC mimic
－ － + －
－ － － +
miR-370-3p
