# Supplementary material for: Deficiency of a novel lncRNA-HRAT protects against myocardial ischemia reperfusion injury by targeting miR-370-3p/RNF41 pathway
Source: Front Cardiovasc Med. 2022 Sep 12;9:951463. doi: 10.3389/fcvm.2022.951463 (PMC9510651; doi:10.3389/fcvm.2022.951463)
Supplement: Supplementary file 3 [file Data_Sheet_1.ZIP › Original Source Data╫ε╨┬░μ/Figure 7/Figure 7G.pptx]

## Slide 1
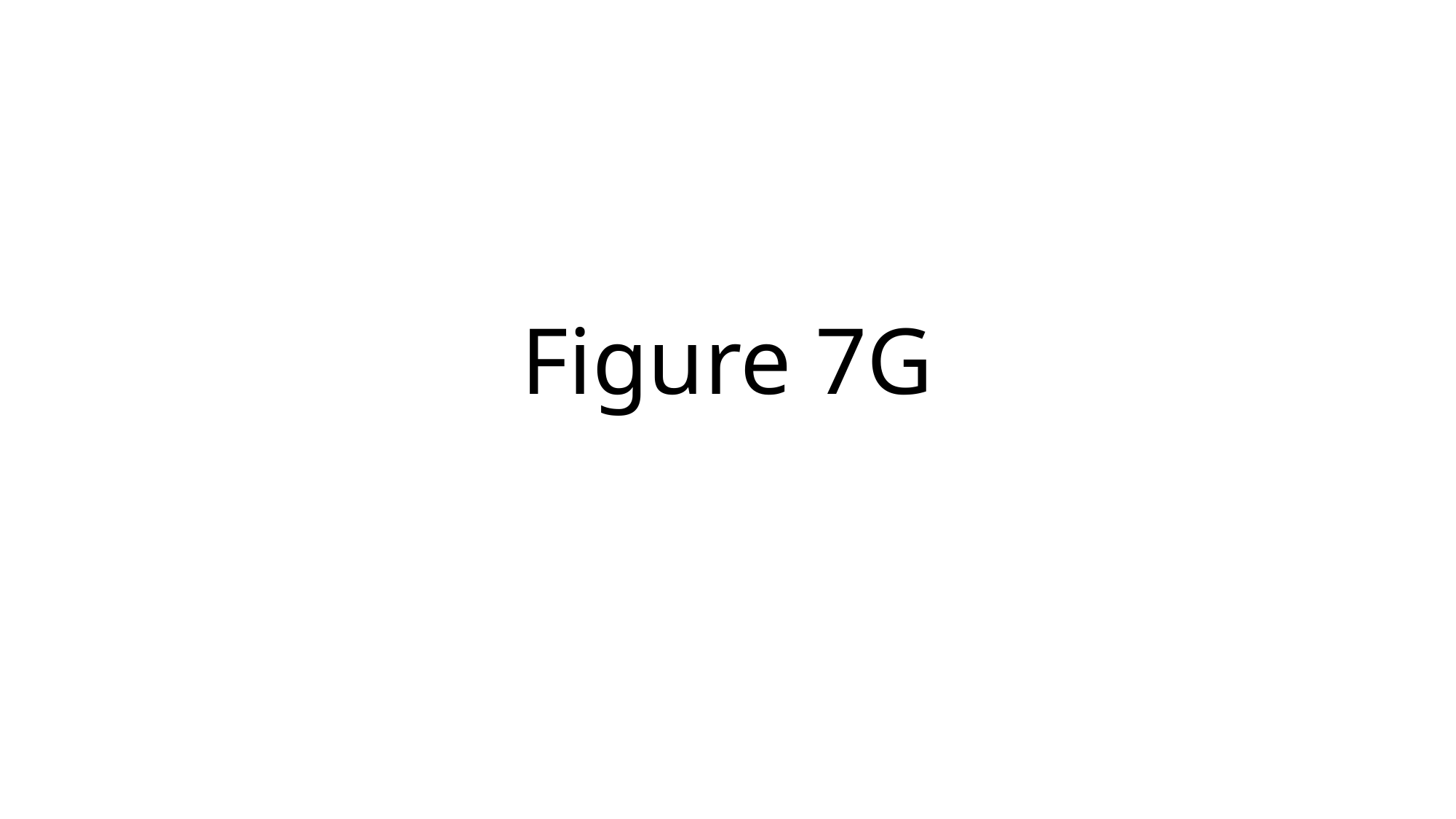

# Figure 7G

## Slide 2
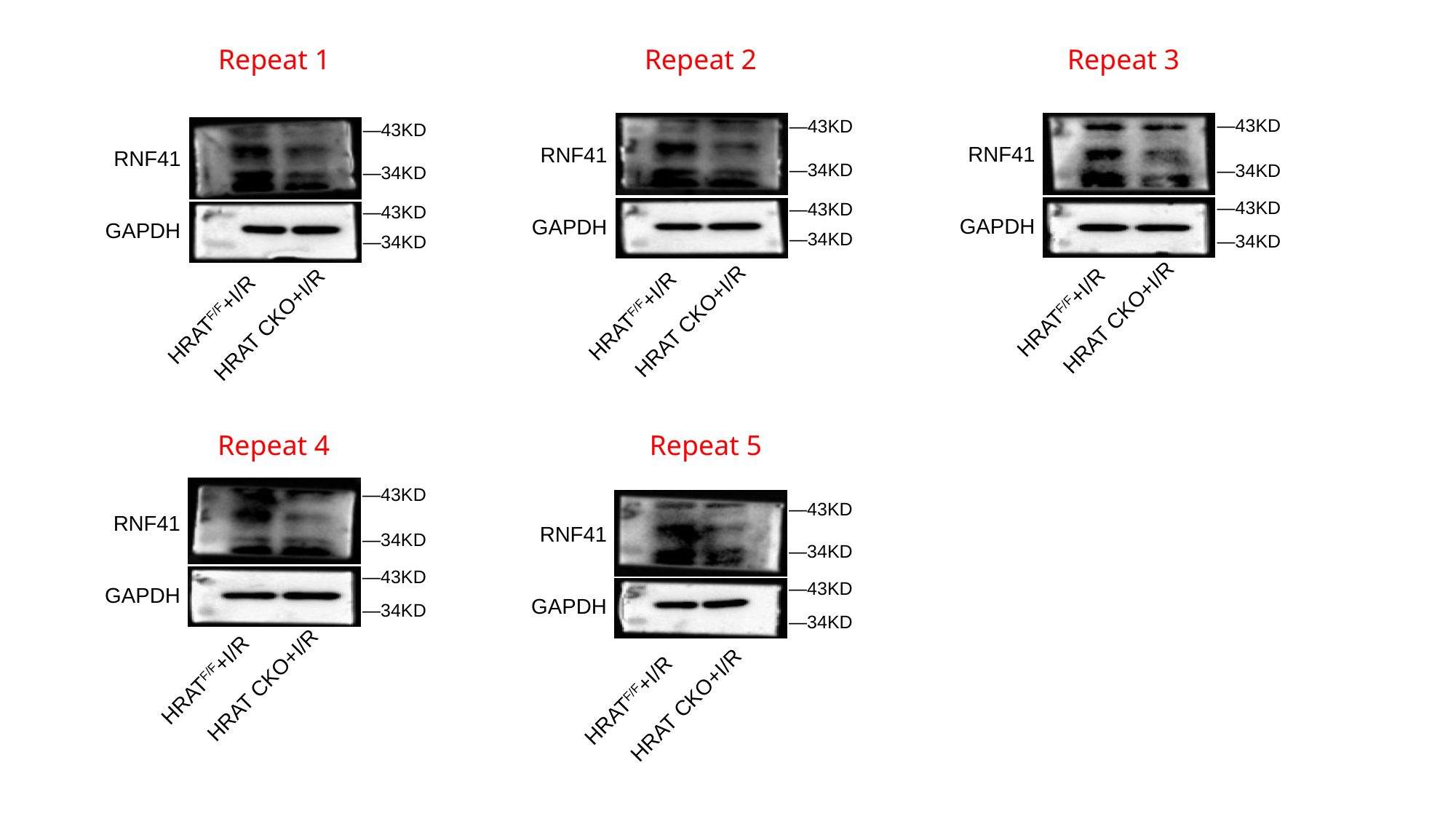

Repeat 2
Repeat 3
Repeat 1
—43KD
—43KD
—43KD
RNF41
RNF41
RNF41
—34KD
—34KD
—34KD
—43KD
—43KD
—43KD
GAPDH
GAPDH
GAPDH
—34KD
—34KD
—34KD
HRATF/F+I/R
HRATF/F+I/R
HRATF/F+I/R
HRAT CKO+I/R
HRAT CKO+I/R
HRAT CKO+I/R
Repeat 4
Repeat 5
—43KD
—43KD
RNF41
RNF41
—34KD
—34KD
—43KD
—43KD
GAPDH
GAPDH
—34KD
—34KD
HRATF/F+I/R
HRAT CKO+I/R
HRATF/F+I/R
HRAT CKO+I/R
